# Liquid Crystal Microcavity Biosensors for Real-Time Liver Injury Monitoring via Whispering Gallery Mode Laser

**DOI:** 10.34133/research.0824

**Published:** 2025-08-05

**Authors:** Jianwei Wang, Yeshuai Song, Xinyu Dou, Jiapeng Sun, Xinghua Yang, Yu Zhang, Zhihai Liu, Yanzeng Li, Hanyang Li

**Affiliations:** ^1^ College of Physics and Optoelectronic Engineering, Harbin Engineering University, Harbin 150001, China.; ^2^ Key Laboratory of In-Fiber Integrated Optics of Ministry of Education, Harbin Engineering University, Harbin 150001, China.; ^3^ Rose-Hulman Institute of Technology, Terre Haute, IN 47803, USA.

## Abstract

Real-time monitoring of liver injury is essential for preserving physiological health. Alanine aminotransferase (ALT) detection is widely regarded as a fundamental approach for the early diagnosis of liver injury. However, existing detection methods often suffer from complex operation, high costs, and limited sensitivity. Here, we introduce a real-time biosensor based on functionalized liquid crystal microcavities with whispering gallery mode (WGM) laser for rapid and sensitive ALT detection. The microcavity functionalized with stearic acid exhibits distinct optical responses to ALT-catalyzed enzymatic reactions across varying concentrations. The high-quality factor of the microcavity obviously enhances its biosensing performance. Simulations reveal variations in the electric field behavior of WGM lasing as the liquid crystal microcavity transitions between radial and bipolar configurations. Further analysis was conducted using experimental WGM spectra and corresponding polarized optical microscopy images. Experimental results demonstrate a strong linear correlation between ALT concentration and reaction time within the range of 0 to 240 U/l. This biosensor exhibits a sensitivity of 0.67 s/(U/l), indicating its potential as a promising approach for early liver injury assays. In addition to in vitro verification, in vivo validation using mouse serum samples further confirms its practical applicability, yielding results consistent with those tested by using commercial assay kits. This method offers a simple, cost-effective, and efficient detection of ALT, underscoring the potential of liquid crystal microcavities for biosensing in liver injury monitoring.

## Introduction

Reliable evaluation of liver function is fundamental for the diagnosis, prognosis, and treatment of hepatic disorders [1]. Alanine aminotransferase (ALT), a well-established biomarker for hepatocellular injury, is critical for clinical assessment [2,3]. Its concentration in serum directly correlates with the severity of liver injury, playing a vital role in the early diagnosis [4], drug-induced hepatotoxicity prevention [5], and postoperative recovery monitoring [6]. Real-time monitoring of ALT levels provides valuable insights into transient liver injury, enabling personalized hepatoprotective interventions. Current ALT detection methods, including electrochemical [7], colorimetric [8], and fluorescence [9], are commonly utilized in clinical assays and provide a reasonable sensitivity. In addition, some emerging strategies such as microfluidics-based platforms [10], surface plasmon resonance sensors [11], and photonic crystal fiber biosensors [12] have also been explored to enhance ALT detection sensitivity. However, these analysis methods often require bulky instruments, multi-step procedures, and lengthy analysis times [13], which severely limit their applicability for real-time clinical scenarios such as intraoperative monitoring and home-based healthcare. Consequently, there is a pressing need for a real-time, convenient, and highly sensitive detection method to address ALT detection in clinical applications.

Recently, whispering gallery mode (WGM) optical microcavities have attracted increasing attention in sensing due to their high *Q*-factor [14,15] and small mode volume [16], which substantially enhance light−matter interactions that improve detection sensitivity and enable real-time sensing. These optical microcavities have been widely applied in low-threshold lasing [17], nonlinear optics [18], high-performance sensing [19], and advanced biosensors for clinical biomarker detection [20–22]. Moreover, the structural and material design flexibility of WGM optical microcavities further opens new possibilities, particularly with the integration of liquid crystal (LC) microdroplets. This combination offers a novel platform for biosensing, leveraging the unique optical anisotropy and tunability of LC molecules [23,24]. The LC microdroplet-based WGM microcavity facilitates the realization of high-efficiency lasing due to its nearly perfect spherical geometry and ultra-smooth surface [25–27], allowing for real-time spectral monitoring, quantitative spectral shifts, and enhanced sensing sensitivity. The foundation of this approach can be traced back to the work of Humar and Muševič [28] on biosensing through spectral analysis, which has since been further refined and developed for a broader range of sensing applications [29–31]. In addition, LC-based WGM microcavities exhibit excellent environmental responsiveness, making them highly suitable for dynamic biochemical sensing, such as detecting variations in pH [32], temperature [33], and biomolecular concentrations [34]. Hence, this platform can serve as an effective strategy for real-time monitoring of ALT in clinical diagnostics.

In this work, we introduce a highly sensitive method for ALT detection utilizing functionalized LC microcavity biosensors via WGM laser. Functionalized materials with environmental responsiveness induce orientation changes of LC molecules within the microcavity. This reorientation changes the refractive index profile of microcavity, resulting in obvious resonance wavelength shifts in the WGM spectra. Structural transitions of LC molecules within the functionalized microcavity were observed in this experiment. The influence of these orientational changes on the electric field distribution of WGM lasing was further analyzed through finite-difference time-domain (FDTD) simulations. Moreover, this biosensor is specifically focused on the ALT concentrations with clinically significant thresholds (40, 80, and 200 U/l) for the accurate assessment of early liver injury. The proposed biosensing method’s simplicity, low cost, real time, and efficiency make it particularly suitable for clinical scenarios requiring frequent ALT assessments in liver health monitoring.

## Results and Discussion

**Fig. 1.**
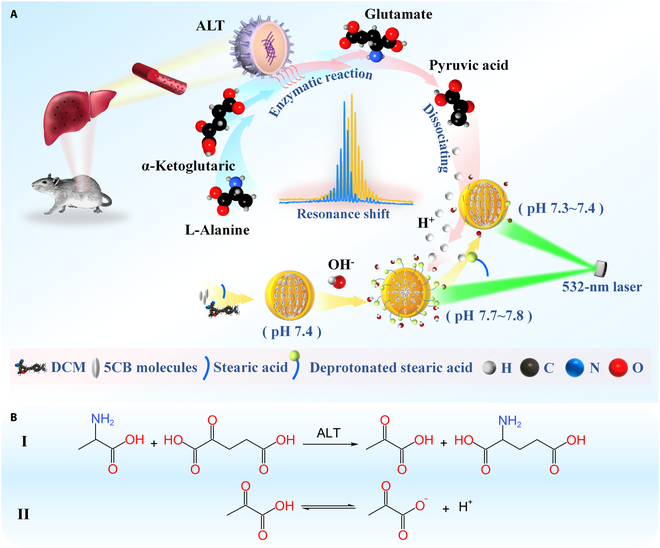
(A) Biochemical sensing principle of ALT detection via WGM lasing. (B) Enzymatic reaction of ALT (I) and dissociation of pyruvate (II).

The biosensing principle of the LC microcavity based on WGMs is illustrated in Fig. 1A. The functionalized LC microcavity was primarily composed of 5CB molecules (4′-pentyl-4′-cyanobiphenyl) and DCM dye [4-(dicyanomethylene)-2-methyl-6-(4-dimethylaminostyryl)-4*H*-pyran]. Besides, stearic acid was introduced as the functional material for surface modification within the microcavity, attributed to its pH-responsive amphiphilic architecture and favorable biocompatibility [35,36]. The molecular structure of stearic acid comprises a long hydrophobic chain and a hydrophilic head group (Fig. S1C) and effectively modulates the orientational behavior of 5CB molecules. The LC microcavity is formed in the phosphate-buffered saline (PBS) aqueous solution because of its hydrophobic property, with 5CB molecules self-assembling into a bipolar configuration within the microdroplet, driven by the confining surfaces. Elevated hydroxide ion (OH^−^) concentrations induce deprotonation of surface-modified stearic acid [35], enhancing its amphiphilic character and driving interfacial self-assembly at the microdroplet interface. This reorganization alters the surface anchoring conditions, which triggers a reorientation transition of 5CB molecules within the LC microdroplet. Subsequently, the long axes of these molecules gradually align radially, resulting in a stable radial configuration. Conversely, elevated hydrogen ion (H^+^) concentrations trigger protonation of the surface-assembled deprotonated stearic acid [36], modulating interfacial anchoring forces to drive 5CB molecular reorientation, ultimately inducing the microcavity to revert to a bipolar configuration. The transition between these 2 configurations induces a marked change in the refractive index of the functionalized LC microcavity, resulting in a noticeable wavelength shift in the WGM lasing spectra.

ALT is a vital biomarker for liver health, released into the bloodstream during liver cell injury. It is a bio-enzyme that catalyzes enzymatic reaction between l-alanine (l-Ala) and α-ketoglutarate (α-KG), facilitating the transfer of the amino group from l-Ala to α-KG [8,9]. This enzymatic process produces the production of pyruvate and glutamate [11,37], as illustrated in reaction I of Fig. 1B. It has been shown that the produced pyruvate dissociates in weakly alkaline aqueous solutions (reaction II in Fig. 1B), releasing hydrogen ions (H^+^) that consequently alter the solution’s pH. Under optimal reaction conditions, the enzymatic reaction rate is directly influenced by the ALT concentration, with the reaction temperature being maintained at approximately 37 °C. The functionalized LC microcavity was prepared in a buffer solution containing l-Ala and α-KG, with the reagent proportions provided in Figs. S2 and S3. The 532-nm laser was used to pump the functionalized LC microcavity to excite WGM lasing during the process of enzymatic reaction. Upon the addition of ALT for catalysis, the resulting increase in H^+^ concentration—released from the dissociation of produced pyruvate—can be monitored in real time via resonance wavelength shifts in the WGM spectra, which allow for the quantification of the variation in the ALT concentration. Additionally, the proposed WGM biosensor allows for rapid, label-free biosensing in this experiment.

**Fig. 2.**
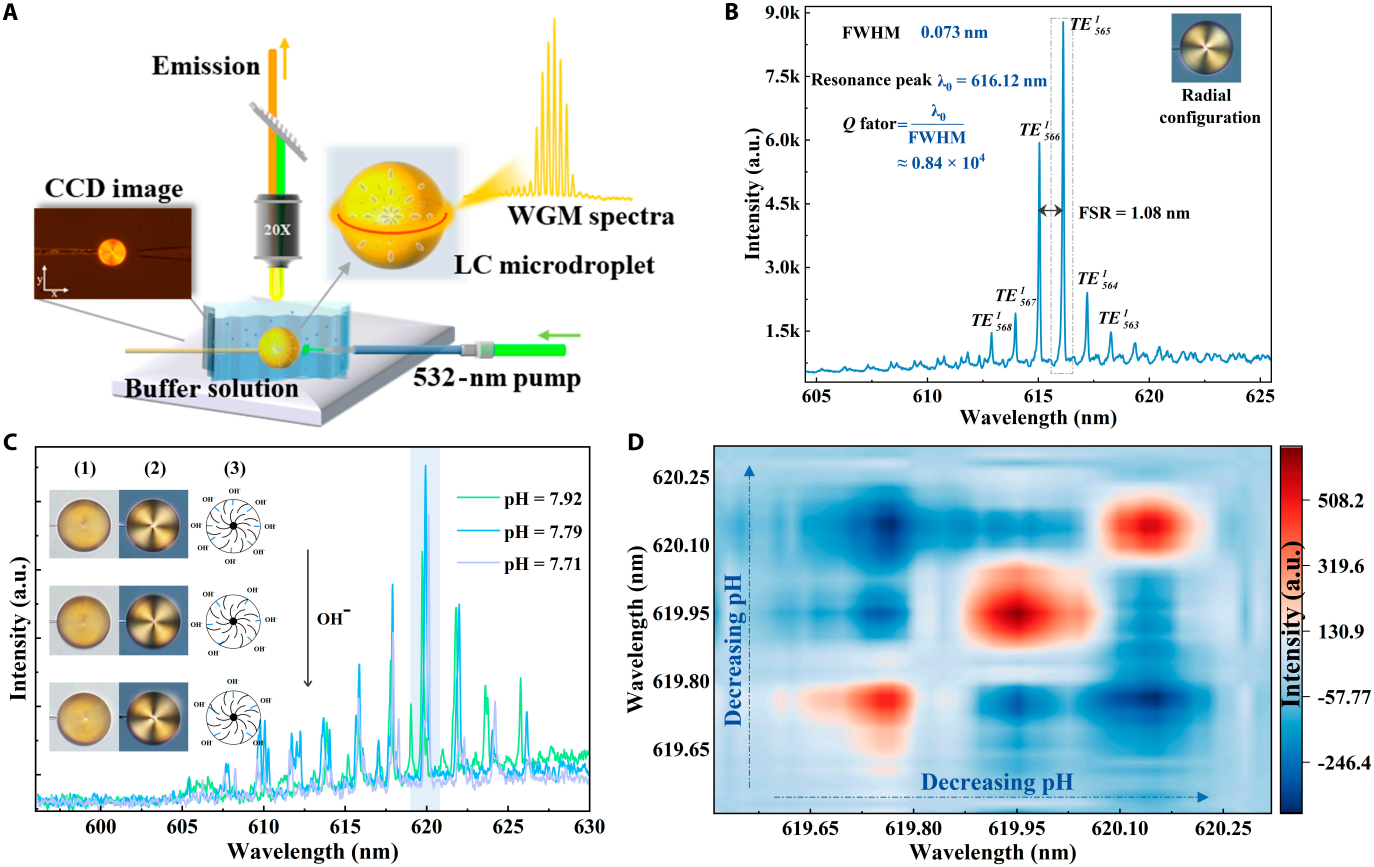
(A) Illustration of the WGM-functionalized LC microdroplet. (B) Typical WGM lasing of a functionalized LC microcavity with radial configuration and its corresponding mode numbers labeled by ${\mathrm{TE}}_{1}^{q}$. The insets show its POM and *Q*-value. (C) WGM spectra for decreasing pH from 7.9 to 7.7. The insets show their microscopic image (1), POM (2), and schematic image (3). (D) 2D correlation spectra of the same mode for decreasing pH.

The schematic diagram of the functionalized LC microcavity using WGM laser is shown in Fig. 2A (the complete experimental setup is provided in Fig. S8). To characterize the optical properties of WGM lasing, the microcavity was pumped using a Nd:YAG pulsed laser (532 nm, 10 Hz, 6 ns). The LC microcavity was generated in a microtube by adjusting the syringe pump in the buffer solution (pH 7.7, 37 °C), enabling effective control over the microcavity size. Furthermore, environmental temperature has a critical role in maintaining enzyme activity, and its influence on the LC microcavity was further investigated in Fig. S5. The WGM lasing spectrum at the radial configuration was collected by a spectrometer with a 0.07-nm resolution, as presented in Fig. 2B. Transverse-electric (TE) modes were observed to dominate in the radial configuration of the LC microcavity. The electric field oscillates parallel to the long axis of the molecules within the microcavity surface in this mode (Fig. S6A) [38], corresponding to the ordinary refractive index (*nₒ* = 1.54). Measured WGM lasing spectrum corresponds to the first-order TE modes, which is demonstrated by the numerical calculation of WGM modes in the Supplementary Materials. The theoretical calculation of the mode numbers shows excellent agreement with the experimentally observed lasing peaks. It was shown that a high *Q*-factor (0.84 × 10^4^) was achieved, calculated based on the spectral half-height width (Fig. S6C). The measured free spectral range (FSR) was in excellent agreement with the theoretical value, calculated by FSR *= λ*^2^/2*πn*_eff_*R* [39]. Furthermore, the lasing threshold characteristics of the LC microcavity was also presented in Fig. S6B.

For traditional LC sensing, its analyzed information is typically derived from the polarized optical microscopy (POM) patterns [40–42], demanding high imaging precision and experimental operation. However, there may be unnoticeable differences in the POMs for subtle detecting changes. As shown in the insets (1 and 2) of Fig. 2C, POM images of PBS buffer solutions with varying pH values (7.92, 7.79, and 7.71, as measured in Fig. S2D) all exhibited radial configurations, with their corresponding grayscale values indicated in Fig. S9A, showing no noticeable changes. In contrast, an obvious red shift in the WGM lasing spectra was observed in the detailed analysis of resonance peaks (Fig. S9B). This is attributed to its higher *Q*-factor and enhanced sensitivity [10,43]. As shown in Fig. 2D, the WGM resonance spectra of the same transmission mode shift to longer wavelength as a result of decreasing pH, highlighting the enhanced sensitivity of the functionalized LC microcavity with WGM laser for biosensing applications.

**Fig. 3.**
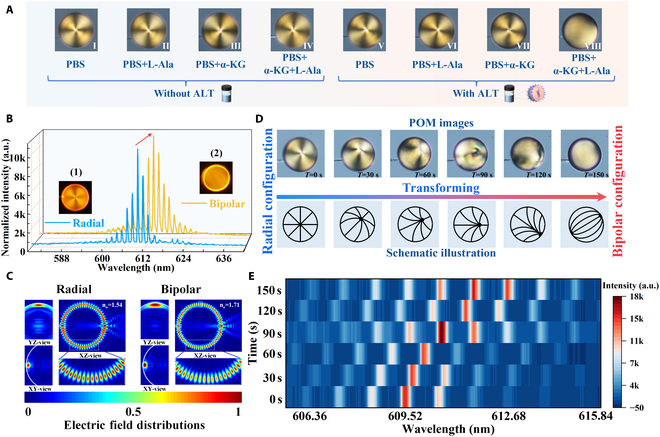
(A) POM patterns of LC microcavities formed in different PBS solution. (I) to (IV) without ALT: (I) only PBS; (II) l-Ala; (III) α-KG; (IV) l-Ala and α-KG. (V) to (VIII) with ALT: (V) only PBS; (VI) l-Ala; (VII) α-KG; (VIII) l-Ala and α-KG. (B) WGM lasing of LC microcavities with radial and bipolar configurations. Insets (1) and (2) show the POMs and (C) corresponding electric field distributions of the radial and bipolar configurations, respectively. (D) POM images with their corresponding schematic illustration of LC microcavity for states from radial to bipolar configuration (recorded after 6 min). (E) WGM spectra recorded from 0 to 150 s.

To ensure optimal enzymatic reaction conditions, the PBS buffer (pH 7.7) was selected as the base solvent at 37 °C, containing 0.1 mM α-KG and 0.167 mM l-Ala (see Fig. S3A and B). The pH variation induced by the enzymatic reaction drives the reorientation of 5CB molecules within the functionalized LC microcavity, as validated by the control experiment shown in Fig. 3A. It was observed that the functionalized LC microcavity transitioned to a bipolar configuration only in the presence of ALT, α-KG, and l-Ala (VIII), while a stable radial configuration in all other conditions (all POM images recorded at 6 min). This indicated that the reorientation of 5CB molecules was driven by the products of the enzymatic reaction. To further validate this hypothesis, WGM lasing spectra were recorded for a 45-μm-diameter LC microdroplet in reaction solutions following the addition of 200 U/l ALT, as depicted in Fig. 3B. It was found that LC microdroplets within this size range could support stable WGM lasing with relatively high *Q*-factors and well-defined spectral characteristics, making it suitable for subsequent spectral analysis. Initially, the microdroplet exhibited a radial configuration (inset 1), with the corresponding WGM spectrum (blue curve). After the addition of 200 U/l ALT, the microdroplet gradually transitioned to a bipolar configuration (inset 2) within approximately 150 s, accompanied by an obvious redshift in the WGM spectrum (orange curve). The resonant spectral shift of the WGM lasing, induced by changes in the aqueous environment surrounding the functionalized LC microdroplet, validates its potential as an enzymatic reaction sensor. Furthermore, the finite difference time domain (FDTD) simulation [44] was performed to analyze the electric field distribution within the LC microcavity for these 2 configurations. For the TE polarization mode, the electric field oscillates parallel to the microdroplet surface. The effective refractive index of the microcavity is determined by the alignment of 5CB molecules. In the radial configuration, the field primarily interacts with the ordinary refractive index (*nₒ*), whereas in the bipolar configuration, it predominantly senses the extraordinary refractive index (*nₑ*). As shown in Fig. 3C, the simulated electric field distributions are displayed in the *x*–*y*, *x*–*z*, and *y*–*z* views. These results reveal distinct variations in field behavior as LC microcavity transitions between radial and bipolar configurations. Accordingly, the evolution of POM images with their relevant schematic illustrations and WGM spectra during the enzymatic reaction was systematically analyzed in Fig. 3D and E. It is observed that the LC microcavity transitions from radial to bipolar configuration, accompanied by a redshift in the WGM spectra, within 150 s in the reaction solution containing ALT. The corresponding shifts in the WGM resonant wavelength at each 30-s interval are shown in Fig. S10. These results demonstrate that WGM lasing in the proposed functionalized LC microcavity can serve as an effective standard for enzyme activity detection, offering a highly sensitive and label-free approach for biosensing.

**Fig. 4.**
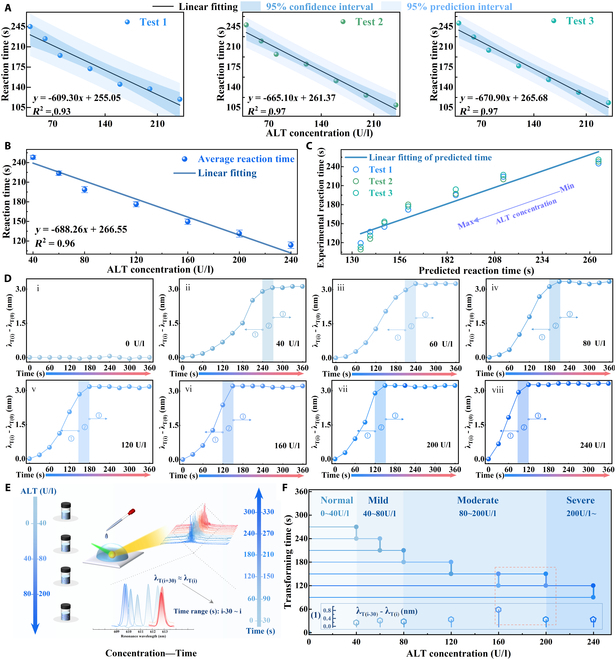
The linear fitting of reaction time with increasing ALT for (A) 3 tests and (B) their average. (C) Relative relation between experimental and predicted reaction time. (D) Spectral responses for different concentrations of ALT: (i) 0 U/l; (ii) 40 U/l; (iii) 60 U/l; (iv) 80 U/l; (v) 120 U/l; (vi) 160 U/l; (vii) 200 U/l; (viii) 240 U/l. (E) Schematic illustration of relationship between ALT concentration and reaction time by WGM spectra. (F) Transforming time with increasing ALT and its corresponding wavelength shift.

Generally, the normal reference range for ALT is typically 0 to 40 U/l, with critical threshold concentrations of 40, 80, and 200 U/l being particularly relevant for the clinical diagnosis of early liver injury [3,7,45]. In this experiment, a fixed volume of 0.02 ml of ALT enzyme was added to each reaction solution. The relationship between ALT concentration and its catalytic reaction time was systematically investigated by evaluating various ALT concentrations (0, 40, 60, 80, 120, 160, 200, and 240 U/l) in 0.5-ml reaction solutions, where the substrates containing l-Ala and α-KG were present in excess. These tests were performed in triplicate for minimizing experimental error. Figure 4A illustrates the linear correlation between reaction time and ALT concentration. It is shown that as ALT concentration increases, the reaction time decreases significantly, which can be attributed to the enzyme’s efficient catalysis of amino group transfer and the rapid production of pyruvate under constant substrate conditions [8,9]. Furthermore, it also presents the confidence and prediction intervals for these 3 tests, meaning good consistency of the experimental data. As shown in Fig. 4B, the average reaction time is plotted as a function of ALT concentration. A linear regression analysis defines the sensitivity as *Δt*/*Δ*[ALT], yielding an average sensitivity of approximately −0.67 s/(U/l). This indicates that for every 1 U/l increase in ALT concentration, the reaction time is reduced by approximately 0.67 s. To further validate the correlation between enzymatic reaction time and ALT concentration, the Michaelis–Menten equation [46] was applied to calculate theoretical reaction times of enzymatic reaction (Fig. S7). Figure 4C shows the correlation between the experimentally measured reaction times (hollow points) and the theoretical predicted values (blue line), where the former is obtained from real-time measurements and the latter is calculated using the Lineweaver–Burk equation [47]. These experimental results exhibit good consistency with the theoretical predictions.

The WGM lasing response is presented in Fig. 4D, with bar markers denoting completed reaction intervals, identified by the wavelength remaining unchanged between adjacent test intervals [λ_T(i+30)_ = λ_T(i)_]. In particular, Fig. 4D (ii) illustrates a continuous redshift of the WGM resonance peak during ALT catalysis, with the wavelength steadily increasing from 0 to 270 s and then stabilizing from 270 to 360 s, which can indicate that the reaction reaches completion within the 240- to 270-s interval. It is found that there is almost no shift in the WGM resonant wavelengths at 0 U/l ALT. In this case, an equal volume of PBS solution was used to replace the enzymatic solution. This result further demonstrates that the spectral response of the functionalized LC microcavity is influenced by changes in the reaction solution owing to enzymatic reaction products. The final wavelength shift corresponds to the saturation of LC molecular reorientation upon complete consumption of α-KG (Fig. S2A). It was also found that the total spectral shift remains consistent, while higher ALT concentrations accelerate the reaction kinetics, leading to a shorter response time. The continuous shifts in WGM spectra corresponding to these concentrations are shown in Fig. S11. The inverse relationship between ALT concentration and reaction time can be determined based on WGM lasing within the functionalized LC microcavity in Fig. 4E, with corresponding completed reaction time range from T(i-30) to T(i). Furthermore, reducing the spectral acquisition interval can further enhance the detection resolution, making the method more suitable for high-precision applications. To improve the accuracy of this proposed method, Fig. 4F presents the analysis of the wavelength shifts (insert 1) at adjacent time intervals within each completed reaction period. At the same reaction completion stage, a higher ALT concentration accelerates the enzymatic reaction rate and leads to a quicker attainment of equilibrium. As a result, the pH change during this phase becomes smaller, causing only a slight shift in the WGM resonance wavelengths. By correlating these spectral shifts with reaction time, the ALT concentration can be rapidly classified as mild (40 to 80 U/l), moderate (80 to 200 U/l), or severe (>200 U/l) liver injury [3,4], thereby facilitating effective evaluation of liver injury severity.

**Fig. 5.**
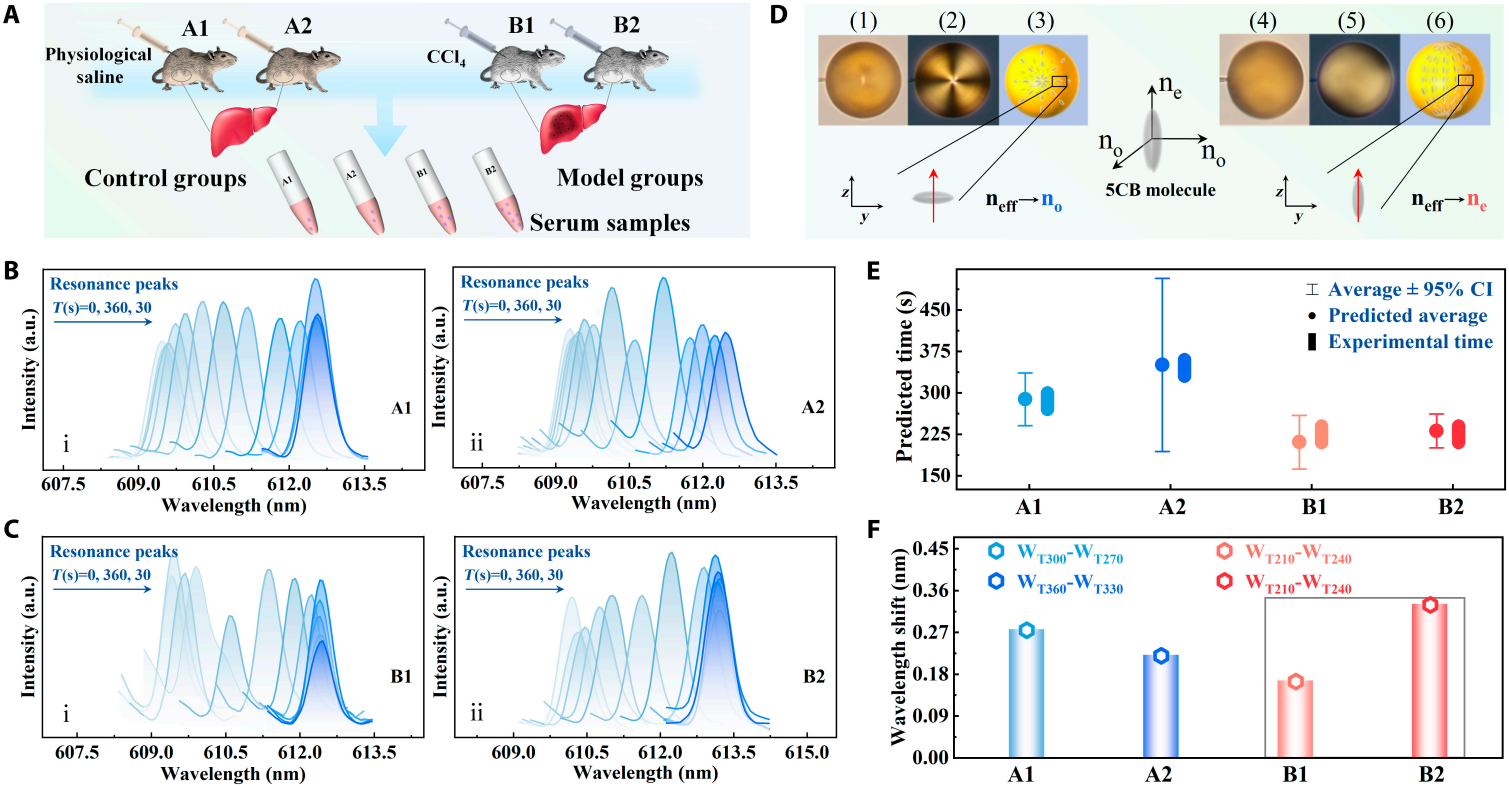
(A) Experimental serum of control and model groups. Resonance peaks of WGM lasing with (B) control and (C) model groups. (D) Transformation of 5CB molecules for radial and bipolar configurations: (1, 4) microscopic image, (2, 5) POM, and (3, 6) geometric diagram. (E) Detecting time of experiments and predicted theoretical time, and (F) corresponding wavelength shifts for the control (A1, A2) and model (B1, B2) groups.

To further validate the applicability of proposed ALT biosensing method, the mouse serum tests were conducted as in vivo experiments for ALT detection. In Fig. 5A, both the control group (normal liver) and the model group (liver injury) consisted of 2 mice each and were maintained under identical conditions. These samples containing ALT were collected from each group, with liver injury induced in the model group using carbon tetrachloride (CCl₄). As provided in Fig. S3C and D, the significantly increased ALT levels (40 U/l < ALT < 80 U/l), observed in the model group (B1 and B2), as compared to the control group (A1 and A2), indicate the successful induction of liver injury. Besides, a controlled experiment was carried out to confirm that the enzymatic reaction was induced by ALT in the serum, using reaction solutions with varying high-pH levels. As shown in Fig. S12A, the observed transition from radial to bipolar configuration within LC microcavity occurred exclusively under pH conditions (pH7.7) favorable to ALT activity, indicating that the molecular reorientation was specifically induced by the ALT-catalyzed reaction. Figure 5B and C presents the WGM lasing spectral resonance peak shifts after adding the serum samples. A redshift was clearly observed within 6 min, attributed to the reorientation of 5CB molecules within the functionalized LC microcavity. The time at which the WGM spectral redshift stabilizes varies among samples and serves as an analysis of the completion of the ALT-catalyzed enzymatic reaction. As shown in Fig. 5D, the short axis of the 5CB molecules responds to a low refractive index (*nₒ*) in the radial configuration. Upon reorganization of the 5CB molecules, their orientation at the surface becomes perpendicular, inducing a change in refractive index of LC microcavity from the ordinary (*nₒ)* to the extraordinary (*nₑ*) refractive index [28,48]. The corresponding WGM lasing response over time and its continuous changes in WGM spectra are also shown in Figs. S12B and S13, respectively. Accordingly, the completed reaction time intervals and the adjacent resonance wavelength shifts after serum addition were analyzed. As shown in Fig. 5E, the experimentally measured reaction times closely match the theoretical predictions, with reaction times for B1 and B2 both falling within the range of 210 to 240 s. However, the adjacent wavelength shift for B1 is smaller than that of B2 in Fig. 5F, reflecting earlier completion of the reaction owing to its higher ALT concentration. The analysis of wavelength shifts within this same period enables further discrimination between their ALT concentrations.

In both in vitro and in vivo ALT detection experiments, the feasibility of biosensing method based on the functionalized LC microcavity using WGM laser was demonstrated. Compared to traditional liver injury diagnose techniques, the proposed method offers notable advantages, especially in identifying ALT concentrations associated with early liver injury, as summarized in Table S1. This biosensor requires fewer substrate materials, which is straightforward to implement, is label-free, and enables rapid response within minutes and real-time monitoring, showcasing its enhanced biosensing performance.

## Conclusion

The proposed method for liver injury monitoring by ALT detection using functionalized LC microcavities offers a promising alternative to traditional biosensing techniques, demonstrating remarkable advantages in terms of sensitivity, simplicity, and real-time monitoring. This biosensing platform based on WGM laser is capable of detecting subtle changes in the refractive index of the LC microcavity, enabling precise, label-free, and cost-effective ALT concentration detection. The strong correlation between ALT concentration and WGM spectral shifts allows for accurate measurement of enzyme activity, positioning this approach as an ideal tool for clinical ALT detection. Furthermore, the system’s capability to operate in both in vitro and in vivo settings, as demonstrated through mouse serum tests, underscores its potential for intraoperative monitoring or home-based healthcare. This biosensing method obviously enhances the sensitivity and accessibility of ALT detection in the range of 0 to 240 U/l, facilitating more efficient liver health monitoring and early liver injury diagnosis.

## Materials and Methods

### Fabrication of functionalized LC microcavity

The functionalized LC microcavity was prepared by mechanically mixing 2.18 g of 5CB (98% purity, UCHEM) with 0.1 wt % DCM (95% purity, Aladdin) and 0.08 wt % stearic acid (C_18_H_36_O_2_, 98% purity, Aladdin). As shown in Fig. S1A, the mixture was thoroughly vortexed for 30 min to ensure uniform dispersion of both DCM and stearic acid within microcavity. The LC microcavity was generated using a syringe pump to precisely control the flow rate of the LC mixture into a tapered microtube with an outer diameter of approximately 5 μm, which was fabricated via the flame tapering method. This approach enables fine tuning of the microcavity size and ensures reproducibility in microcavity formation (Fig. S1B).

### Preparation of mouse serum

The mouse serum samples were collected from a carbon tetrachloride (CCl₄)-induced liver injury model. Eight-week-old male mice were randomly divided into a control group and a liver injury model group (*n* = 2 per group). Mice in the model group received a single intraperitoneal injection of 1% CCl₄ at a dose of 5 ml/kg to induce acute hepatocellular injury, while control mice were injected with an equal volume of physiological saline. After 48 h, whole blood was collected into pro-coagulant tubes, allowed to clot at room temperature for 2 h. The samples were centrifuged at 3,000 rpm for 10 min, and the supernatant serum was harvested for analysis. ALT levels were quantified using a commercial ALT (ALT/GPT) colorimetric assay kit (Nanjing Jiancheng Bioengineering Institute, China) according to the manufacturer’s instructions. Both the control and liver injury model groups included 3 replicate measurements per mouse (*N* = 6). The model group showed significantly elevated ALT levels compared to the control group [significance level: ***(*P* < 0.001)], confirming the successful induction of liver injury, as illustrated in detail in Fig. 3C and D.

### Experiment setup and procedures

As shown in Fig. S8, the pumping light source was employed by frequency-doubled pulses of a Nd:YAG pulsed laser (wavelength: 532 nm, frequency: 10 Hz, pulse duration: 6 ns, pump spot size: 6 mm), and then the pumping lasing was injected into a tapered optical fiber tip through multimode optical fiber via the 1,064-nm filter and a focusing system. The desired-sized functionalized LC microcavity was produced using a microtube by adjusting the injection speed and the operating time of the syringe pump. The buffer solution was applied onto a polymethyl methacrylate (PMMA) substrate, serving as the host medium for the LC microcavity. The lasing emission from the LC microcavity was collected using a 20× (numerical aperture = 0.5) objective. A 532-nm filter was used to remove the pump light, and the lasing emission was directed through a beam splitter to a spectrometer (HiperS-320i, Zolix, China) with an 1,800 lines/mm grating (0.07 nm spectral resolution, 40.3 nm span) and a charge-coupled device (CCD) camera (DP21, Olympus, Japan), with the linear polarizer placed in the optical path to acquire polarizing optical microscopy (POM) images. Besides, the temperature of the buffer solution could be controlled using a heating platform (minimum regulation value of 0.1 °C) and monitored in real time with a temperature probe (Fluke, resolution 0.1 °C) to ensure an optimal reaction environment.

### Simulation of WGM lasing within LC microcavity

The simulation model, comprising a microsphere and a 3-dimensional (3D) parabolic model, was designed to replicate the geometry of the LC microcavity and the tapered optical fiber, as illustrated in detail in Fig. S4.

## Data Availability

All data relevant to this work are available in the manuscript and Supplementary Materials. The data are available from the corresponding author upon reasonable request.
