# Supplementary material for: Liquid Crystal Microcavity Biosensors for Real-Time Liver Injury Monitoring via Whispering Gallery Mode Laser
Source: Research (Wash D C). 2025 Aug 5;8:0824. doi: 10.34133/research.0824 (PMC12324788; doi:10.34133/research.0824)
Supplement: Supplementary 1 — Supplementary Text Figs. S1 to S13 Table S1 [file research.0824.f1.docx]

**Supplementary Materials**

Liquid crystal microcavity biosensors for real-time liver injury monitoring via WGM laser

Jianwei Wang^1,2^, Yeshuai Song^1^, Xinyu Dou^1^, Jiapeng Sun^1^, Xinghua Yang^1,2^, Yu Zhang^1,2^, Zhihai Liu^1,2^, Yanzeng Li^3^*, and Hanyang Li^1,2^*

*^1^College of Physics and Optoelectronic Engineering, Harbin Engineering University, Harbin 150001, China;^2^Key Laboratory of In-Fiber Integrated Optics, Ministry Education of China, Harbin Engineering University, Harbin 150001, China; ^3^Rose-Hulman Institute of Technology,* *Terre Haute, IN 47803, USA.*

E-mail: hanyang_li@qq.com; liy2@rose-hulman.edu.

**Contents**

**1. Experimental materials and methodology** S-1

**Fabrication of functionalized LC microcavity**S-1

Figure S1 (a) The processing of fabrication for functionalized LC microcavity.S-1

Figure S1 (b) Sizes of LC microcavityS-1

Figure S1 (c) Illustration of amphiphilic characteristics of stearic acidS-1

**Preparation of reaction solution**S-2

Figure S2 (a) Schematic illustration of the ALT-catalyzed transamination reaction.S-2

Figure S2 (b) The POM images of functionalized LC microcavities with different pHS-2

Figure S2 (c) Three independent tests were conducted at pH 7.3-7.7S-2

Figure S2 (d) Reproducibility of pH measurements for PBS solutions with different pHS-2

Figure S3 (a) POM images with different concentrations for α-KG S-3

Figure S3 (b) POM images with different concentrations for L-Ala S-3

Figure S3 (c) Serum samples of mice S-3

Figure S3 (d) ALT concentrations of serum samples in control and model groups.S-3

**2. Theoretical support and analysis** S-4

**Simulation of WGM lasing within LC microcavity** S-4

Figure S4 Simulation structure of LC microcavity S-4

**Impact of temperature on LC microcavity** S-5

Figure S5 Temperature testing of LC microcavity S-5

**Numerical calculation of WGM modes in LC microcavity** S-6

Figure S6 (a) Schematic illustrations of the electric field for TE and TM mode. S-6

Figure S6 (b) Threshold curves of LC microcavity. S-7

Figure S6 (c) The calculation method of *Q* factor based on the spectral half-height width S-7

**Theoretical analysis of enzyme-catalyzed reaction**  S-7

Figure S7 Reaction time of ALT at varying concentrations and theoretical calculation S-8

**3. Supplementary figures and table** S-8

Figure S8 The schematic diagram of experiment setupS-8

Figure S9 (a) Gray level histogram of POMs for LC microcavity formed in decreasing pH S-9

Figure S9 (b) Variation of WGM lasing resonance peaks for decreasing PH. S-9

Figure S10 Variations of WGM-resonant wavelength S-9

Figure S11 WGM spectra for ALT concentrations:0-240U/LS-10

Figure S12 POMs of LC microcavities with mice serum samples and wavelength shiftsS-11

Figure S13 WGM spectra of ALT concentrations for serum samples S-11

Table S1 Comparison of ALT detection methodsS-12

**References**  S-13

**1. Experimental materials and methodology**

**Fabrication of functionalized LC microcavity.** As shown in Figure S1a, the functionalized LC microcavity was prepared by mechanically mixing 5CB with DCM and stearic acid ^1^. The mixture was further stirred and ultrasonicated for 30 minutes to ensure complete dissolution and homogeneous distribution of DCM and stearic acid within the LC microcavity.


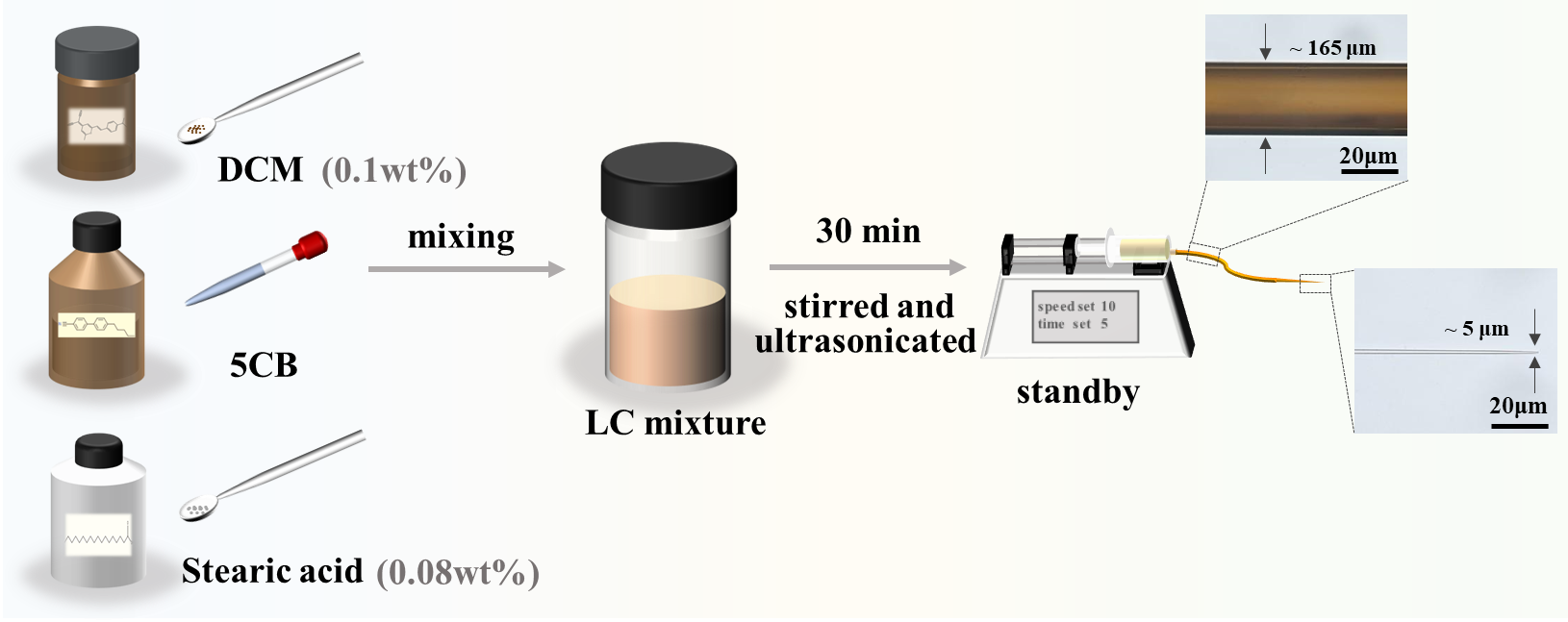


**Figure S1. (a)** The processing of fabrication for functionalized LC microcavity.

Stearic acid is selected as the functionalization material due to its amphiphilic nature, which possesses a hydrophobic tail and a hydrophilic head group. As shown in Figure S1c, in alkaline (OH⁻) solutions, the carboxyl group is deprotonated, rendering the molecule amphiphilic. However, in acidic (H⁺) solutions, the carboxyl group remains protonated, and the molecule behaves predominantly as hydrophobic. It can promote the orderly radial alignment of 5CB molecules in an alkaline aqueous solution, attributed that pH increase facilitated the deprotonation of carboxylic acids^2^, triggering a transition in 5CB molecular orientation from planar to homeotropic anchoring at the microcavity surface. The LC microcavity, with a nearly perfect spherical geometry and smooth surface^3^, was formed by 5CB microdroplets owing to surface tension in the solution. A syringe pump was used to precisely control the flow rate of the liquid crystal mixture into a tapered microtube, thereby generating LC microcavities of different sizes. As shown in Figure S1b, this method exhibited good reproducibility.

**
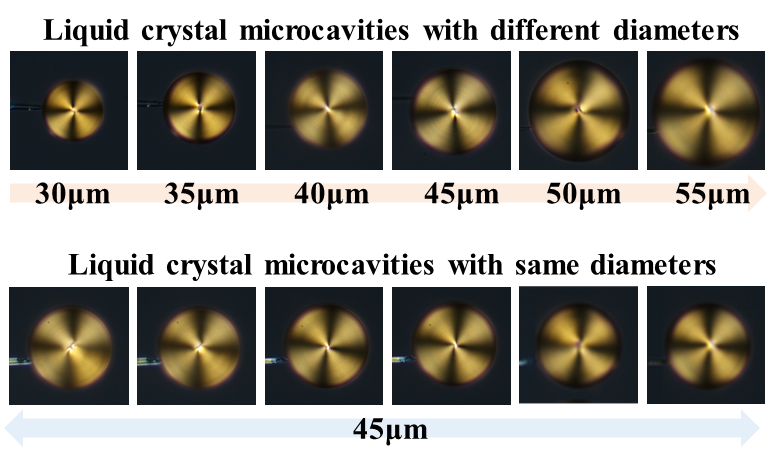
**
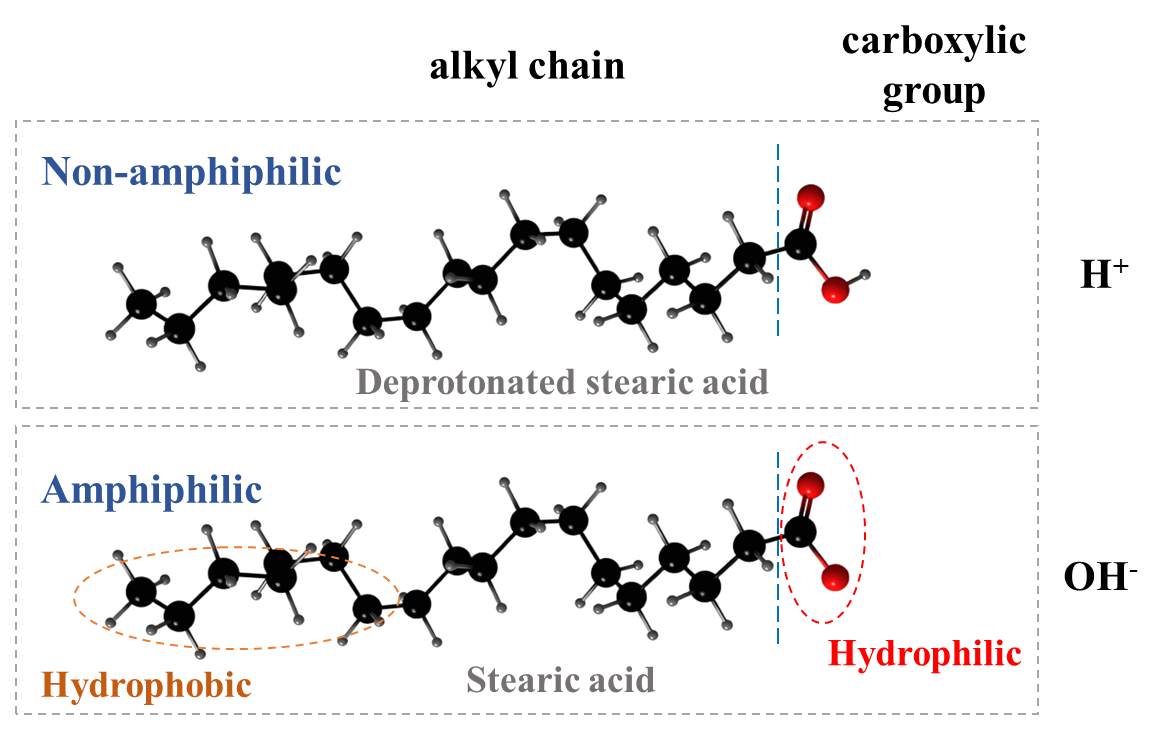


**Figure S1. (b)** Sizes of LC microcavity. **(c)** Illustration of amphiphilic characteristics of stearic acid.

**Preparation of reaction solution.** Owing to the catalytic specificity of alanine aminotransferase (ALT), it catalyzes the transamination between L-alanine (C₃H₇NO₂) and α-ketoglutarate (C₅H₆O₅), transferring the amino group from L-alanine to α-ketoglutarate acid and thereby generating glutamate (C_5_H_9_NO_4_) and pyruvic acid (C_3_H_4_O_3_), which is illustrated in Figure S2a. While ALT accelerates the reaction rate, the final product concentrations are governed by the initial substrate amounts. The buffer solution consists of PBS solution (Yuanye) supplemented with L-alanine (L-Ala, HPLC≥98%, Yuanye) and α-ketoglutarate acid (α-KG, 99% purity, Aladdin). ALT (from porcine heart, Yuanye), the primary enzyme detected in this experiment, exhibits optimal enzymatic activity under conditions of 36–37°C and pH 7.0–8.0^5^.

**
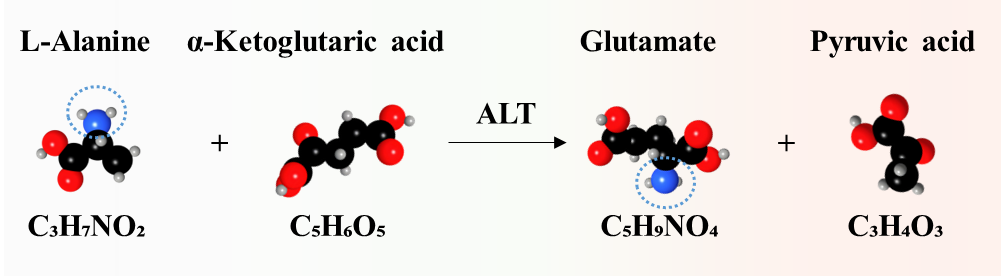
**

**Figure S2.** **(a)** Schematic illustration of the ALT-catalyzed transamination reaction.


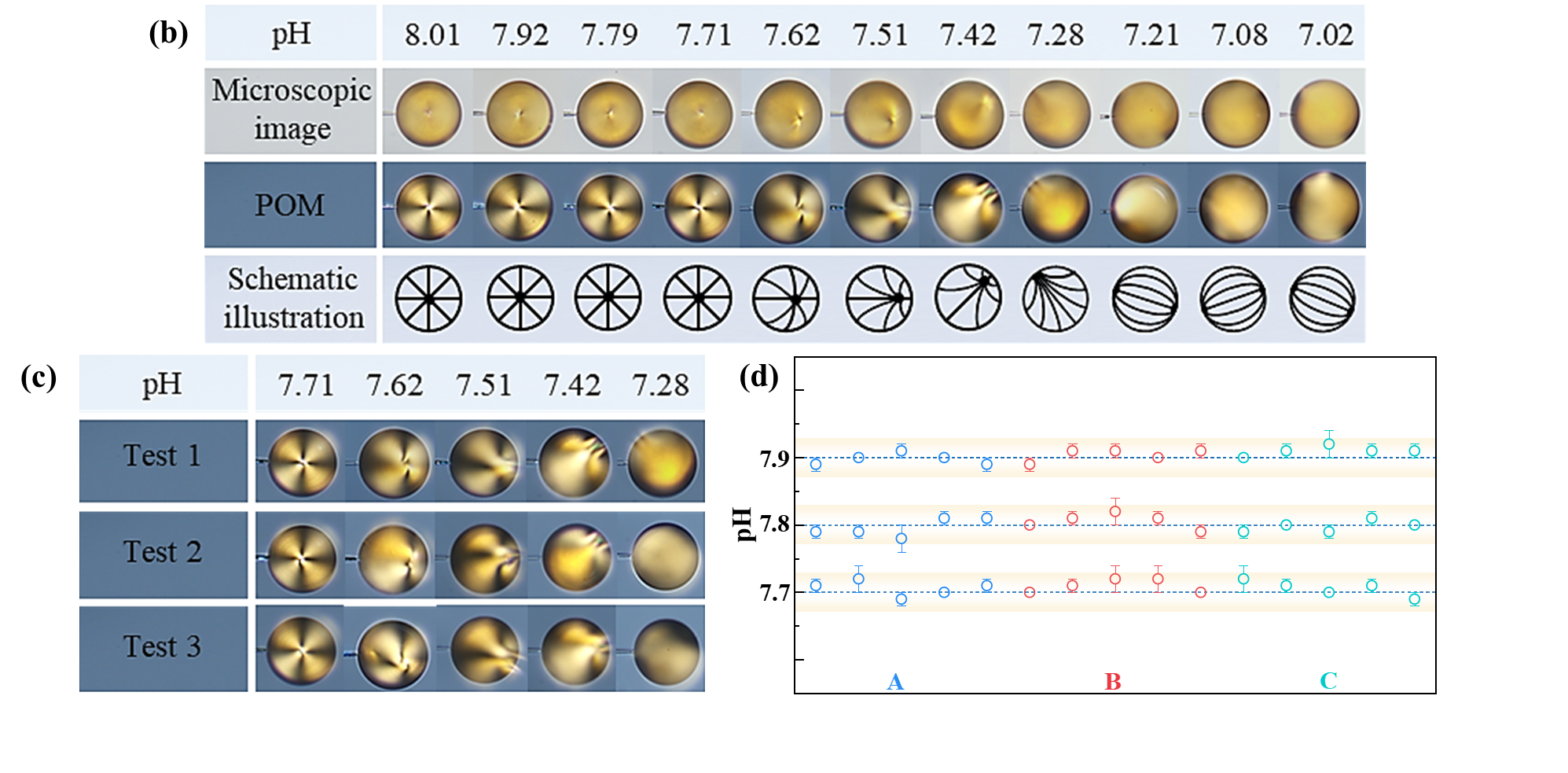


**Figure S2.** **(b)** The POM images of 45μm functionalized LC microcavities in PBS buffer solutions with different pH from 7.0 to 8.0 (recorded after 6 min). **(c)** Three independent tests were conducted at pH 7.3-7.7. (Scale bars 20 μm). **(d)** Reproducibility of pH measurements for PBS buffer solutions with different pH values (7.9 7.8, and 7.7)

To determine the pH range for the deprotonation of stearic acid in functionalized LC microcavity, the pH of the PBS buffer was adjusted incrementally from 7.0 to 8.0 in 0.1-unit steps. A calibrated commercial pH meter was used to ensure accurate pH measurements, which was calibrated using a standard two-point calibration method with calibration buffers of pH 4.00 and pH 9.00. During measurement, the pH electrode was immersed in the PBS solution, gently stirred, and allowed to stabilize for approximately 30 seconds before recording the reading. As shown in Figure S2b, the POM images revealed that functionalized LC microcavities induced molecular rotation within the range of pH 7.3–7.7, thus the initial buffer solution was set to pH 7.7. Then three independent experiments were conducted by adjusting the pH from 7.7 to 7.3 for ensuring reproducibility, and the consistent results are illustrated in Figure S2c. Figure S2d presents the pH measurement results of 7.7, 7.8, and 7.9 independently repeated by three different experimenters (A, B and C).

**
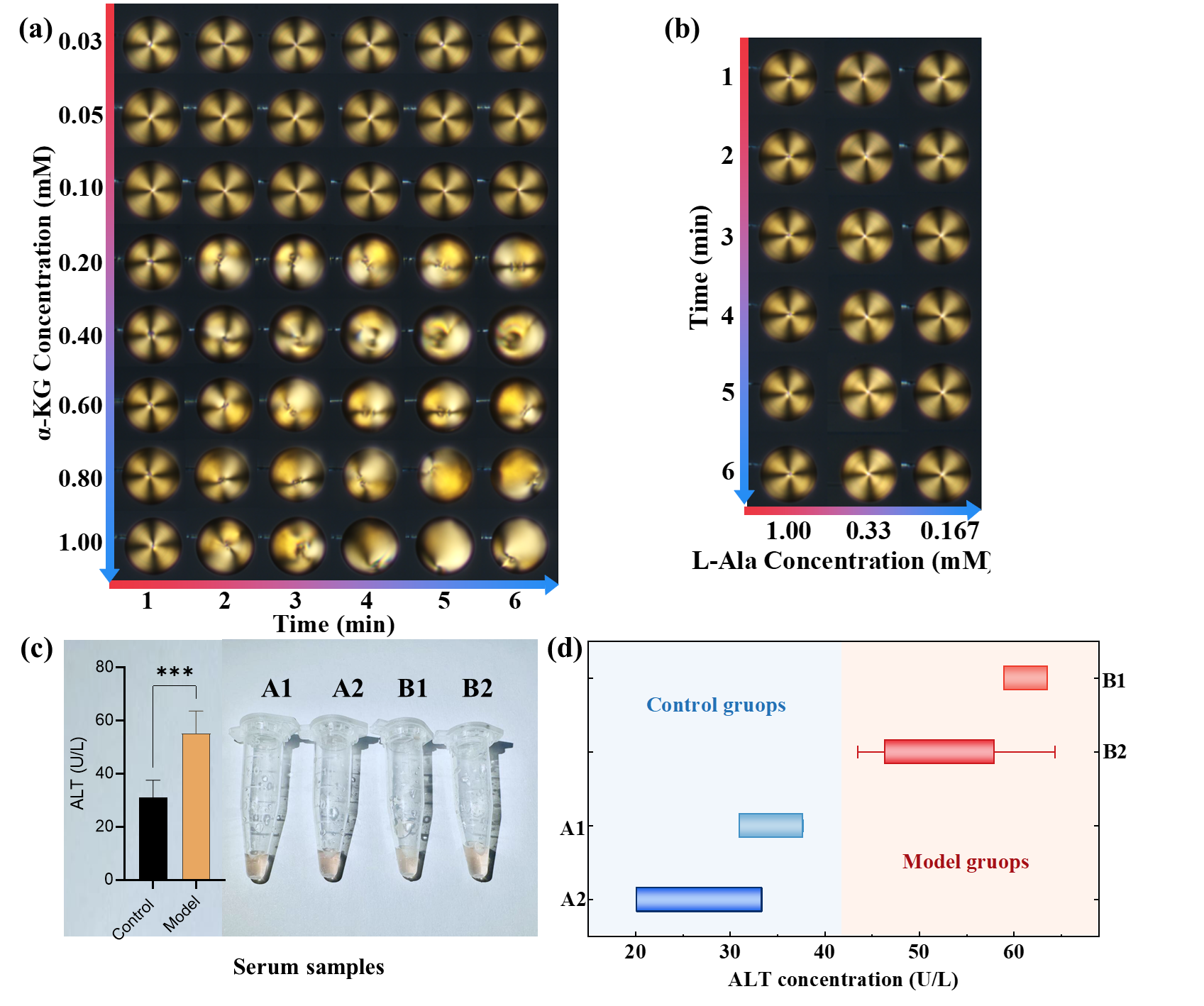
**

**Figure S3.** The POM images of functionalized LC microcavities in PBS solutions (pH 7.7) with different concentrations for **(a)** α-KG and **(b)** L-Ala (Scale bars 20 μm). **(c)** Serum samples of mice and **(d)** ALT concentrations of serum samples in control and model groups.

Figures S3a and S3b further demonstrate the optimization of substrate concentrations (α-KG and L-Ala) through POM imaging captured from 1 to 6 minutes. The findings indicated that high concentrations of α-KG released H⁺ ions in PBS at pH 7.7, thereby lowering the pH and diminishing the anchoring effect of stearic acid on the functionalized LC microcavity surface. Based on these observations, the concentrations of α-KG and L-Ala were optimized to 0.1 mM and 0.167 mM, respectively. Besides, the mouse serum was conducted to evaluate the feasibility of proposed method for the vivo experiments. As shown in Figure S3c, the ALT concentrations of the control (A1, A2) and model groups (B1, B2) correspond to the reference ranges for normal (0–40 U/L) and mildly elevated ALT levels (40–80 U/L), respectively, confirming the successful establishment of the liver injury model, as further supported by Figure S3d. We gratefully acknowledge Enable Biotechnology (shanghai) Co., Ltd. (Shanghai, China) for providing the mouse serum samples and assisting with sample testing.

**2. Theoretical support and analysis**

**Simulation of WGM lasing within LC microcavity.** To further elucidate the WGM lasing behavior within the LC microcavity, numerical simulations were performed using FDTD Solutions. In these simulations, the LC microdroplet was modeled with a diameter of 2.25 μm, and a tapered optical fiber was employed to efficiently excite and couple the whispering gallery modes, which is approximated by a 3D parabolic model. The alignment of 5CB molecules dictates the effective refractive index within the microcavity. When arranged radially, the light primarily probes the ordinary refractive index (nₒ = 1.54); in contrast, the bipolar alignment allows the field to interact predominantly with the extraordinary refractive index (nₑ = 1.71). The simulation model, presented from a perspective view, is illustrated in Figure S4(a). Electric field monitors were placed in the X–Y, X–Z, and Y–Z planes to capture the spatial distribution of the electromagnetic field, as illustrated in Figure S4(b). These simulation results clearly reveal the variations in WGM lasing behavior between the radial and bipolar configurations of the LC microcavity.


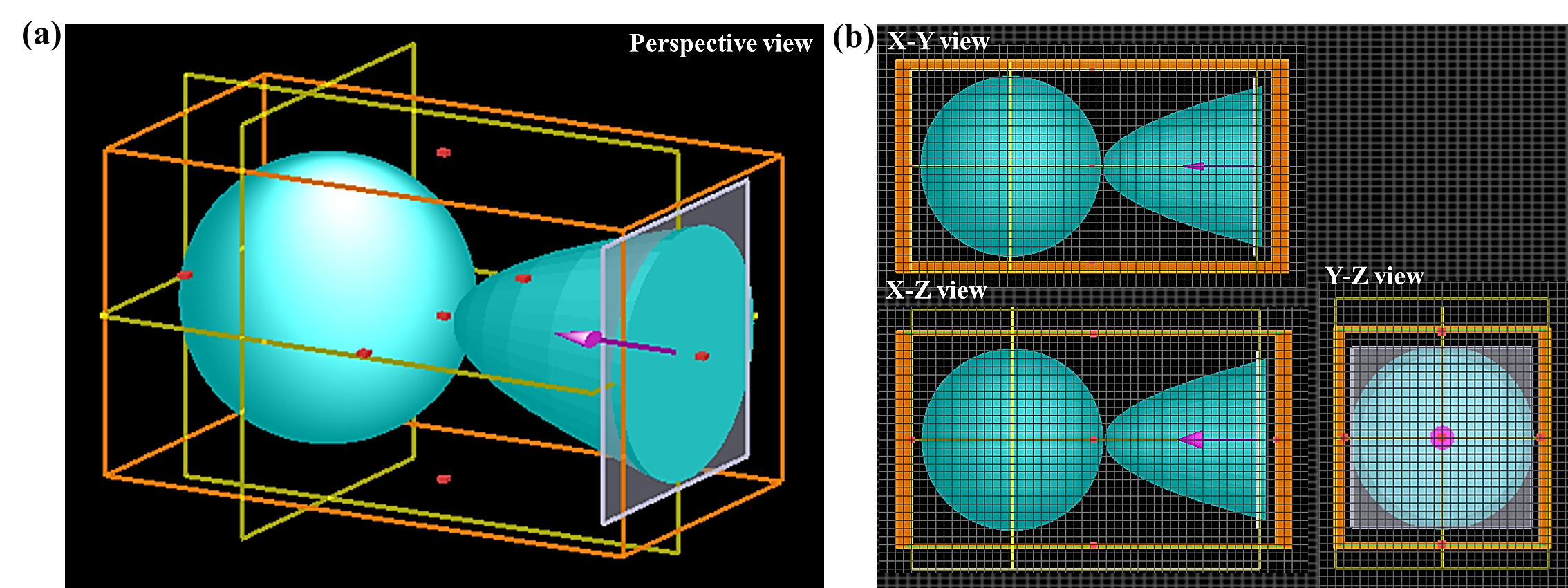


**Figure S4.**  The simulation structure of LC microcavity: (a) Perspective view; (b) X-Y view, X-Z view and Y-Z view.

**Impact of temperature on LC microcavity.** As presented in Figure S5a, the temperature stability of the heating stage was evaluated at set points of 36°C, 37°C, and 38°C. Real-time temperature measurements, recorded using a temperature probe at 30-second intervals over a 6-minute period, demonstrated effective temperature control. The corresponding WGM spectra at these temperatures were acquired through five independent tests, and the resonance peak shifts were systematically analyzed. Figure S5b reveals a slight blue shift in the WGM resonance peaks, as indicated in the inset. The thermally induced resonant wavelength shift (Δ𝜆) can be quantitatively expressed as follows^6^:

$\Delta\lambda=\lambda_{0}\left(\frac{1}{n}\frac{dn}{dT}\Delta T+\frac{1}{D}\frac{dD}{dT}\Delta T\right)$ (S1)

where *dn/dT* represents the thermo-optic coefficient, describing the rate of change of the refractive index with temperature, and *D^-1^dD/dT* corresponds to the thermal expansion coefficient, characterizing the relative dimensional change of the LC microcavity with temperature.


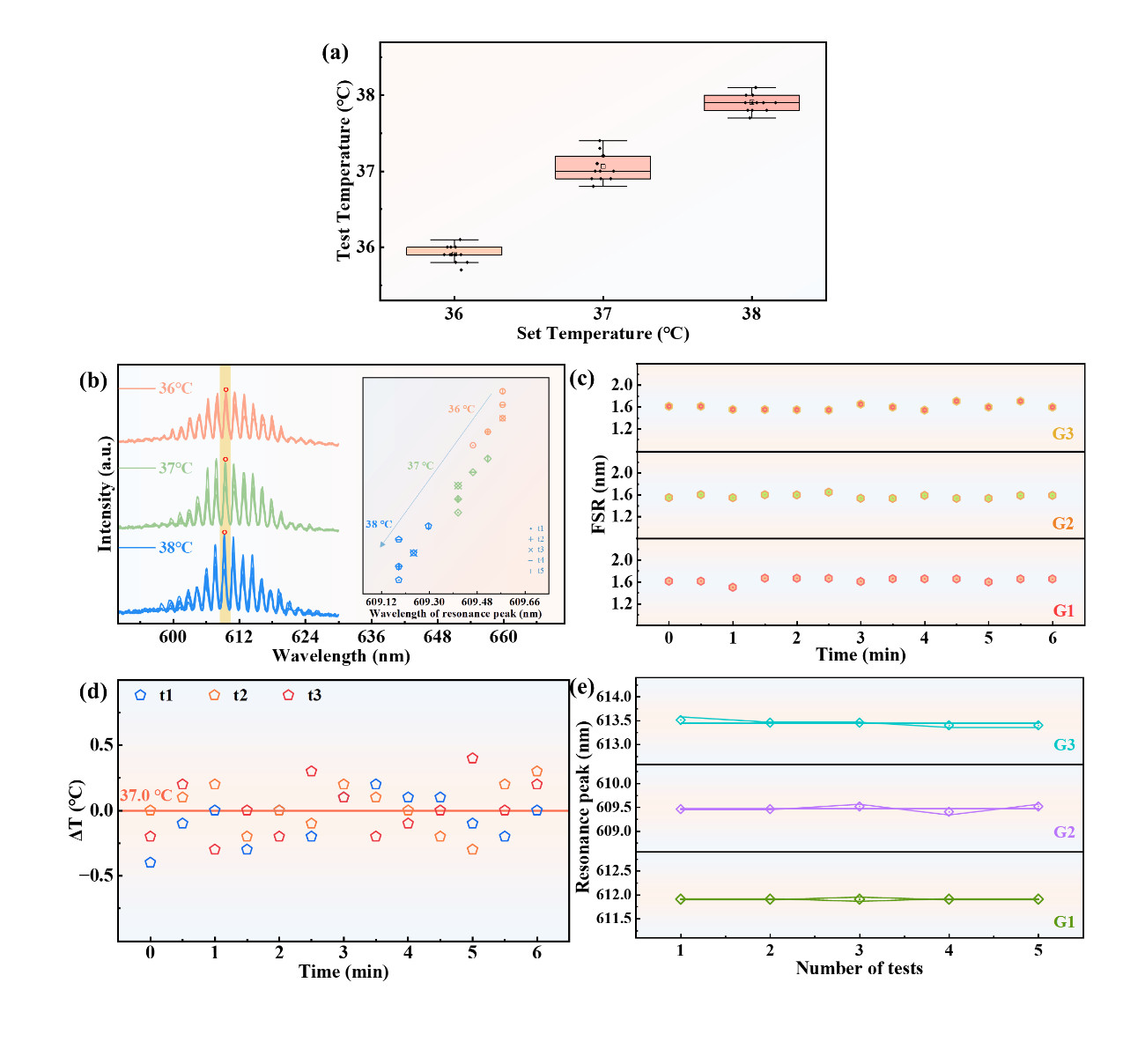


**Figure S5.** **(a)** Testing temperatures at set points of 36°C, 37°C, and 38°C. **(b)** WGM spectra for temperature at 36°C, 37°C, and 38°C (five tests). **(c)** the FSR of the LC microcavities in 6 minutes (three groups). **(d)** the temperature deviation (*ΔT=T-37°C*) at 37°C in 6 minutes (three tests). **(e)** the variations in WGM resonance peaks at 37°C (three groups).

The LC microcavity size was analyzed over a 6-minute period through three independent tests in Figure S5c, the results demonstrate that the free spectral range (FSR) of the LC microcavity exhibited negligible variation. To further evaluate temperature fluctuations at 37°C, three additional tests were conducted over the same time frame. The results, shown in Figure S5d, revealed a maximum temperature deviation (*ΔT*) of less than 0.4°C. Figure S5e illustrates the variations in WGM resonance peaks at 37°C, compared to wavelength shifts caused by external environmental changes, the slight blue shift caused by thermal effects was minimal and can be deemed negligible. All tests were repeated 3 or 5 times to ensure the reliability of the results and to minimize random error.

**Numerical calculation of WGM modes in LC microcavity.** For the spherical WGM cavities, the lasing modes with different azimuthal mode numbers can be approximately determined using the following equation^7,8^:

$\lambda^{-1}=\frac{1}{2\pi rn_{c}}\left[l+\frac{1}{2}-\alpha_{q}(\frac{l}{2})^{1/3}-\frac{\chi n_{r}}{\sqrt{n_{r}^{2}-1}}+\frac{3\alpha_{q}^{2}}{20}(\frac{l}{2})^{-1/3}-\frac{\alpha_{q}}{12}(1+\frac{2\chi n_{r}(2\chi^{2}-3n_{r}^{2})}{\sqrt{(n_{r}^{2}-1)^{3}}})(\frac{l}{2})^{-2/3}\right]$ (S2)

where *l* represents the angular mode number of WGM modes, *λ* is the resonant wavelength of WGM and *r* is the radius of LC microcavity. The refractive index of LC microcavity and the surrounding medium are denoted by *n_c_* and *n_s_*, with the relative refractive index *n_r_* defined as the ratio *n_c_*/*n_s_*. *α_q_* is the roots of the Airy function, where *q* indicates the radial mode number of WGM modes and *q*=1 in this calculation. The parameter *χ* takes values of *χ*=1 for transverse electric (TE) modes and *χ*=1/*n^2^* for transverse magnetic (TM) modes, distinguishing the polarization states of the transmission modes.


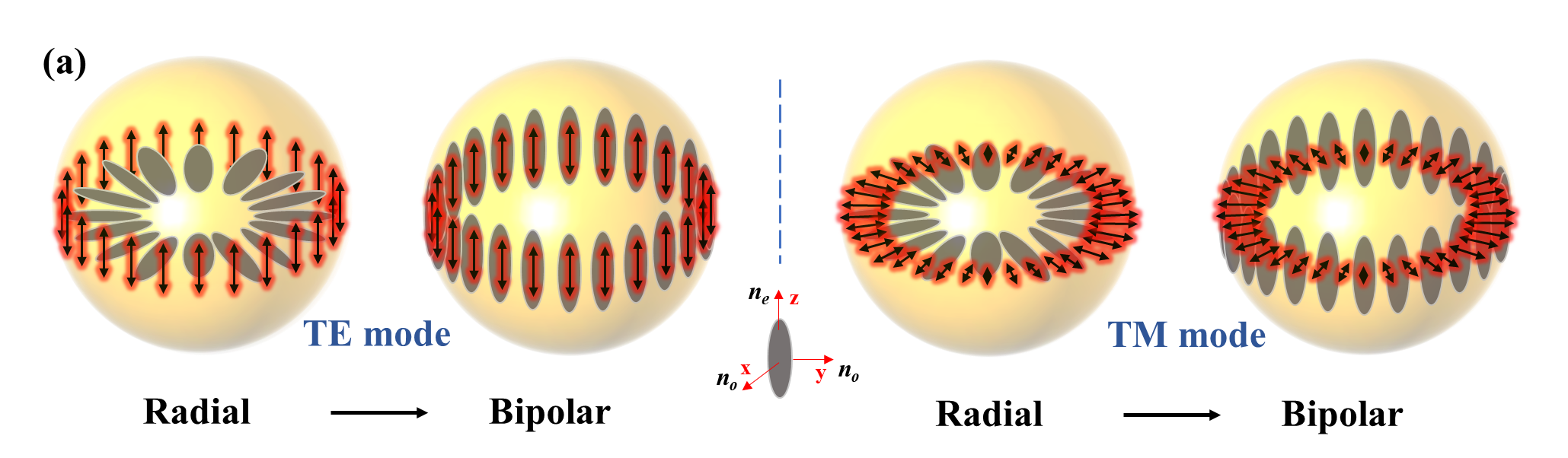


**Figure S6.** (a) Schematic illustrations of the electric field oscillation patterns within the LC microcavity transitioning from radial to bipolar configuration: TE mode and TM mode.

These two types of WGM modes (TE and TM) exhibit distinct polarization states within the microcavity and correspond to different effective refractive indices. As shown in Figure S6a, the TE mode has its electric field oriented perpendicular to the molecular long axis, and consequently senses the much lower ordinary refractive index (*n_o_*). Conversely, the TM mode features a radially oscillating electric field that interacts with the liquid crystal's long molecular axis, thereby experiencing the higher extraordinary refractive index (*nₑ*). In this study, the LC microcavity undergoes a configuration transition from radial to bipolar alignment. This reorientation of the liquid crystal molecules increases the effective refractive index sensed by the TE mode, leading to a red shift in the WGM lasing spectra. The experimentally measured WGM lasing peaks correspond to the first-order TE modes, with azimuthal mode numbers ranging from 563 to 568 (q = 1), which is calculated by Equation (2). When the experimental parameters are substituted into the equation, the theoretically calculated resonant wavelengths for the TE modes show excellent agreement with the experimentally observed lasing peaks, confirming the mode assignment and the sensitivity of WGM lasing to molecular orientation.

To illustrate the occurrence of lasing in the WGM microcavity, photoluminescence spectra of the LC droplet were recorded under varying pump pulse energies. In Figure S6a, the spectral intensity exhibits a nonlinear dependence on the pump energy, confirming the onset of lasing behavior. The lasing threshold was determined to be approximately 0.87 μJ. When the pump pulse energy exceeds this threshold, an obvious light ring emerges around the lasing microdroplet (the inset in Figure S6b), which explains the circulating WGM lasing along the droplet’s surface. Besides, the quality (*Q*) factor within microcavity can be analyzed by WGM spectra (Figure S6c), which is based on the full width at half maximum (FWHM) of the lasing peaks^9,10^, and its calculation method is as follows.

 (S3)

**Figure S6.** (b) Threshold curves of LC microcavity. (c) The calculation method of *Q* factor based on the spectral half-height width.

**Theoretical analysis of enzyme-catalyzed reaction.** ALT, as a catalytic enzyme, accelerates the reaction rate and product formation when its concentration increases, provided that substrate levels are maintained in excess. The substrate concentrations were adjusted to exceed the saturation point of ALT enzyme activity to ensure sufficient reaction substrates during the experiments. The theoretical reaction time of ALT at varying concentrations was calculated based on experimental data using the Michaelis-Menten equation^11^, which was then used to derive the corresponding reaction time. The reaction rate (*v*) and reaction time (*t*) demonstrates an inverse relationship, and the reaction rate increased with substrate concentration in Figure S7a. Three independent tests were conducted to ensure data reliability. In Figure S7b, the experimental data were transformed into the Lineweaver-Burk equation and fitted linearly^12^. The fitting results yielded K_m_ and V_max_ with a R^2^ of 0.91. Additionally, Figure S7c presents the coefficient of variation across different concentrations.


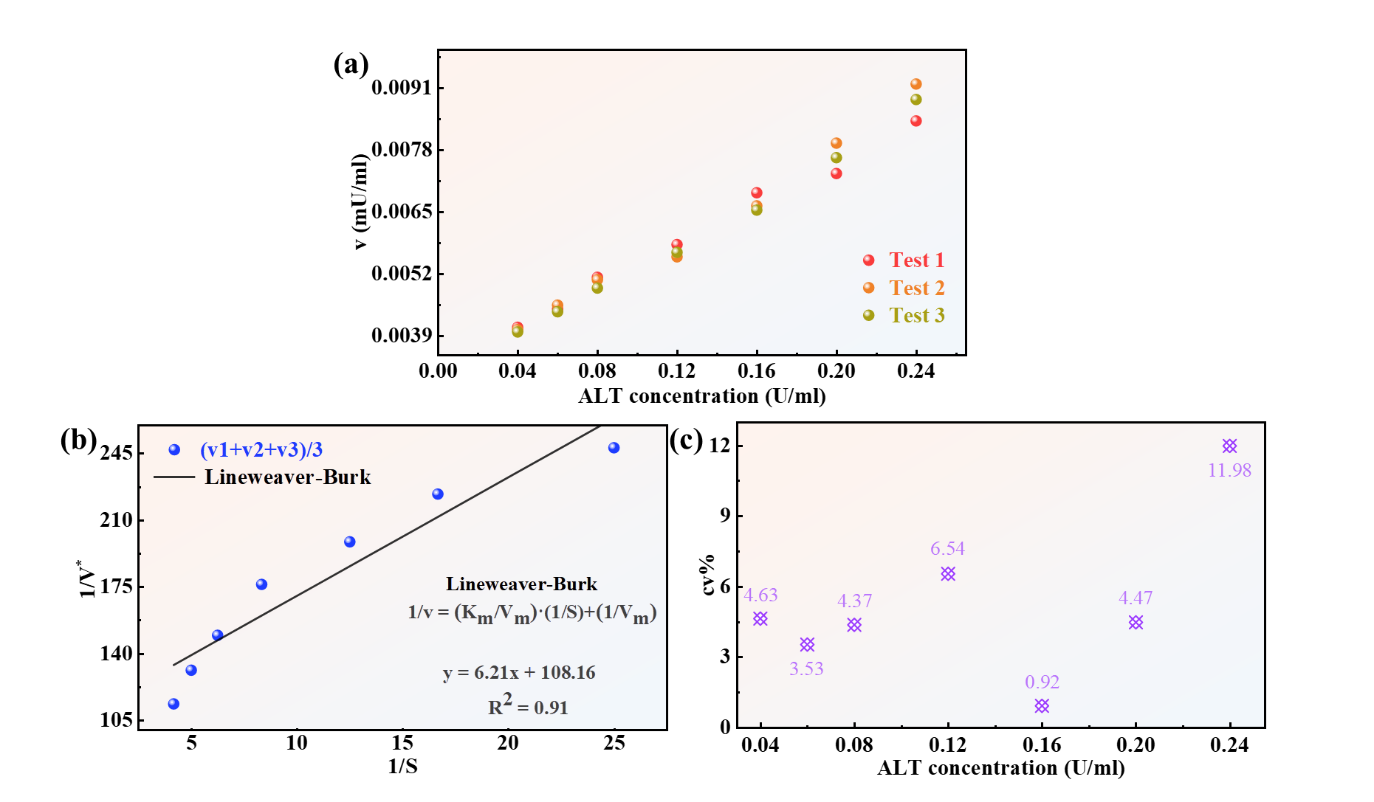


**Figure S7.** **(a)** The testing reaction time of ALT at varying concentrations (three tests). **(b)** the linear fitting of Lineweaver-Burk. **(c)** the coefficient of variation for reaction time.

**3. Supplementary figures and table**


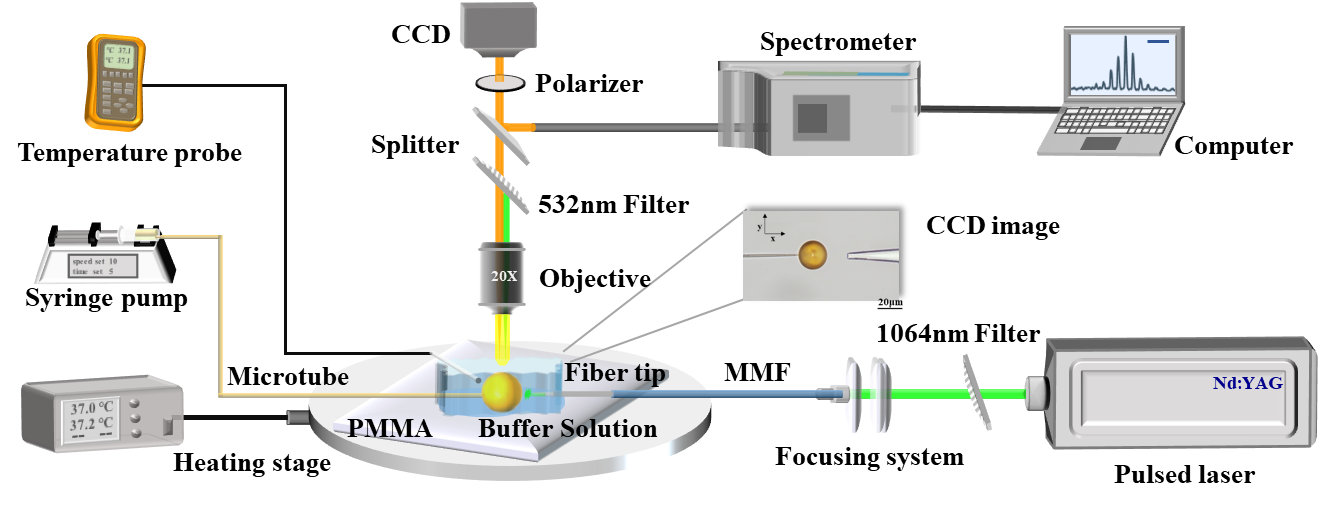


**Figure S8.** The schematic diagram of experiment setup.

**
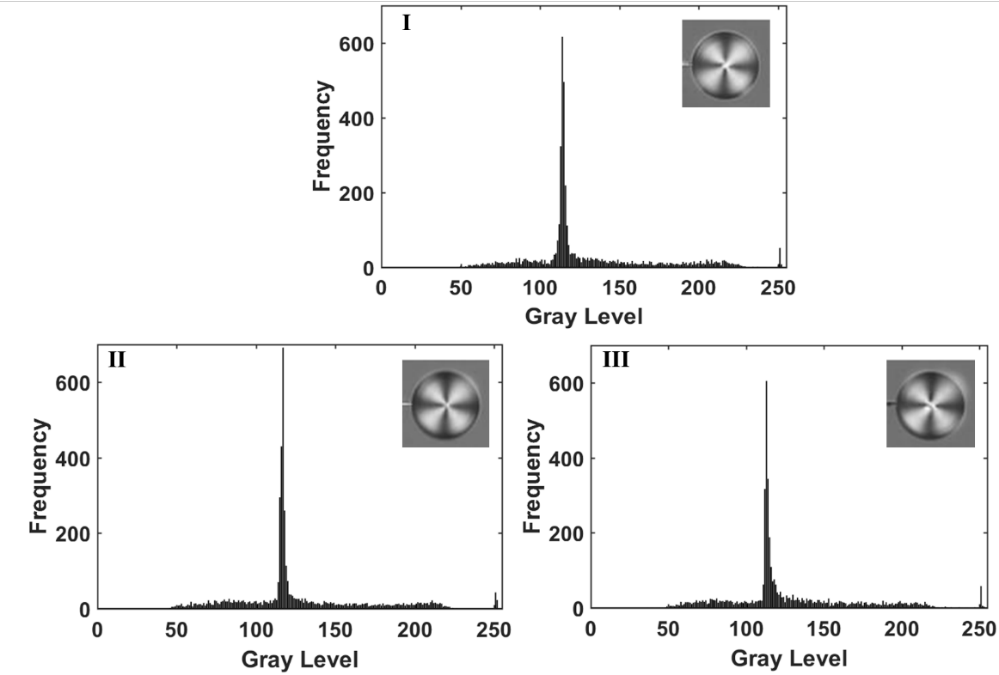
**

**Figure S9.** (a) Gray level histogram of POMs for LC microcavity formed in decreasing pH solution: (I) 7.92; (II) 7.79 and (III) 7.71. The insets show their corresponding grayscale-images.

**Figure S9.** (b) Variation of WGM lasing resonance peaks for decreasing PH.


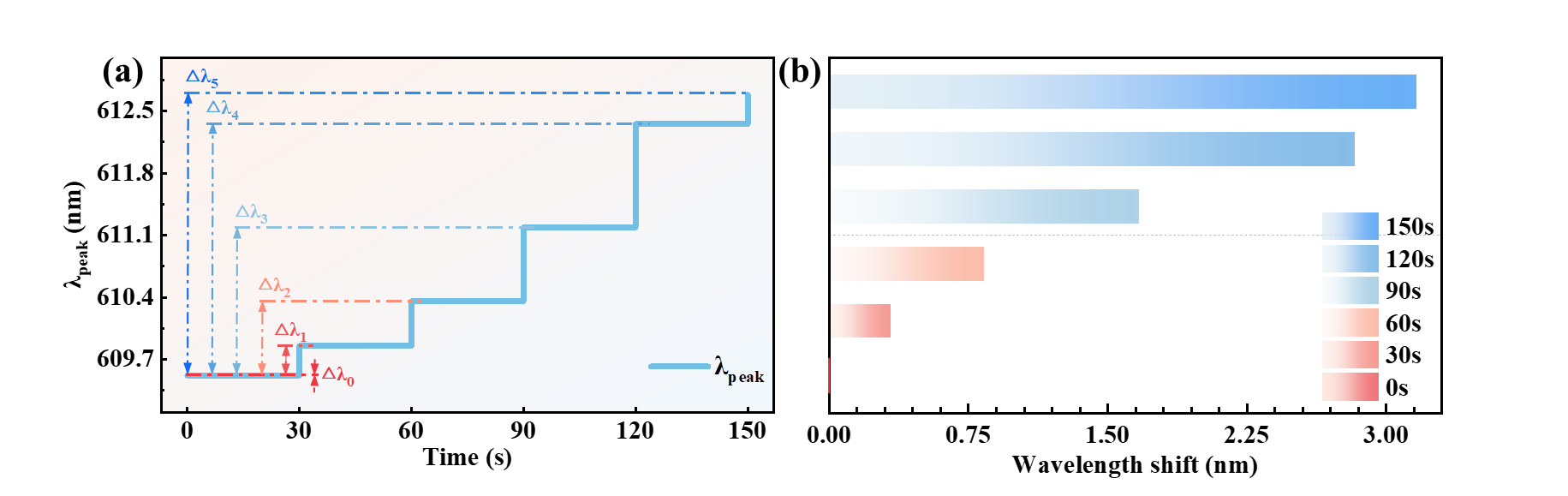


**Figure S10.** **(a)** Variations of WGM-resonant wavelength for reaction time from 0s to 150s. **(b)** The wavelength shifts of resonance peak, relative to the initial time point (0s).


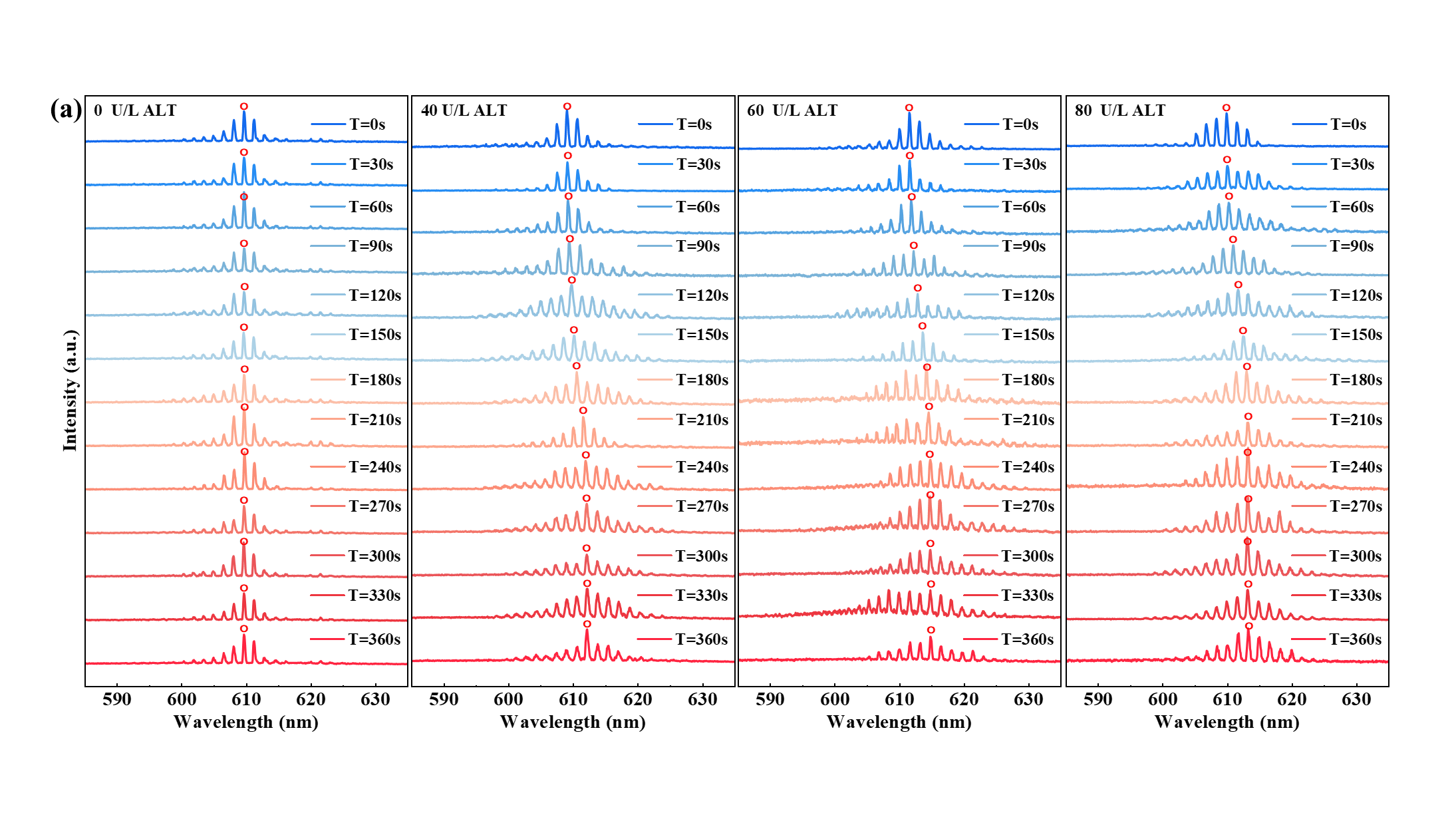


**Figure S11.** **(a)** WGM spectra for ALT concentrations: 0 U/L, 40 U/L, 60 U/L, 80 U/L.


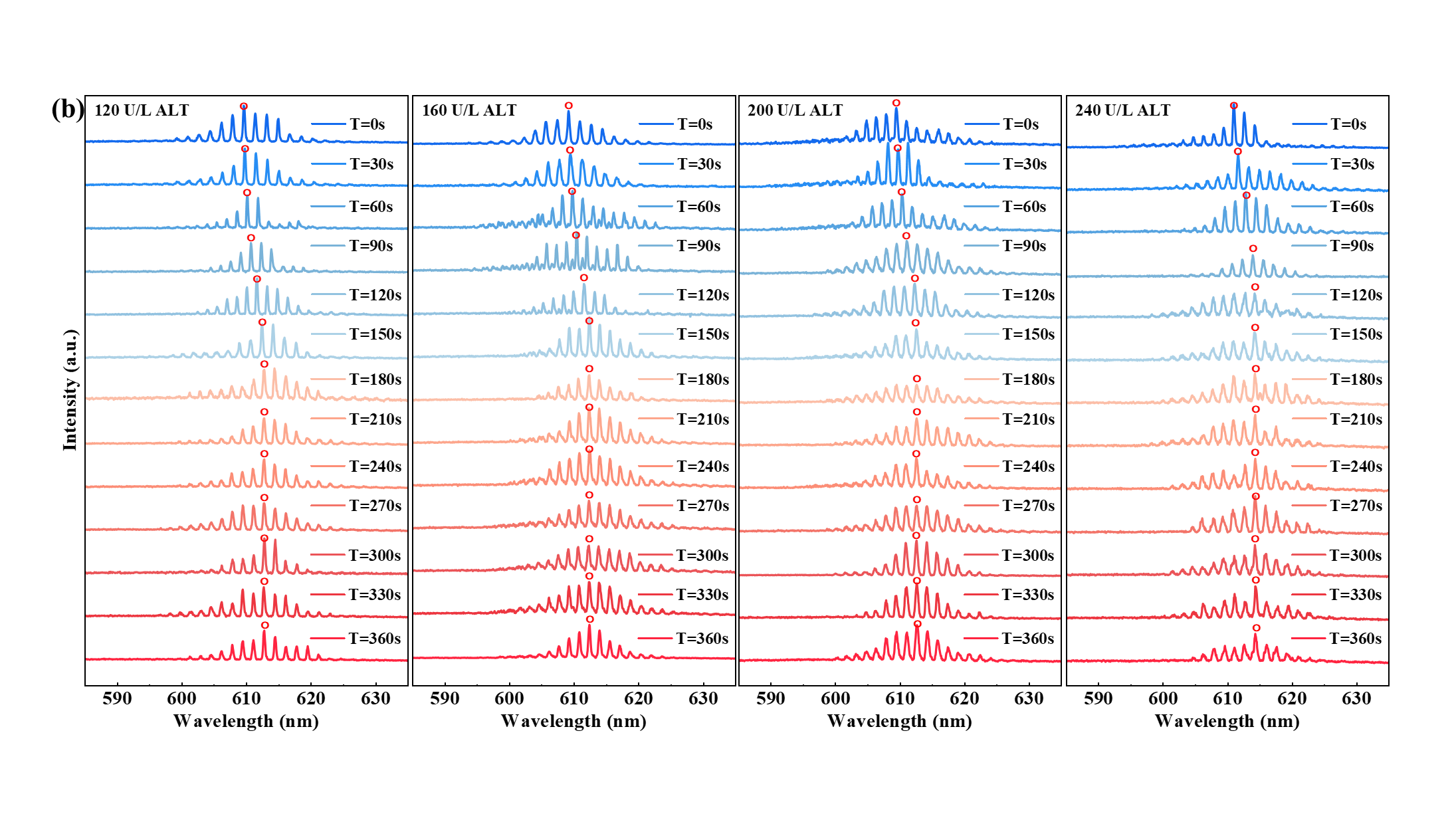


**Figure S11.** **(b)** WGM spectra for ALT concentrations: 120 U/L, 160 U/L, 200 U/L, 240 U/L.


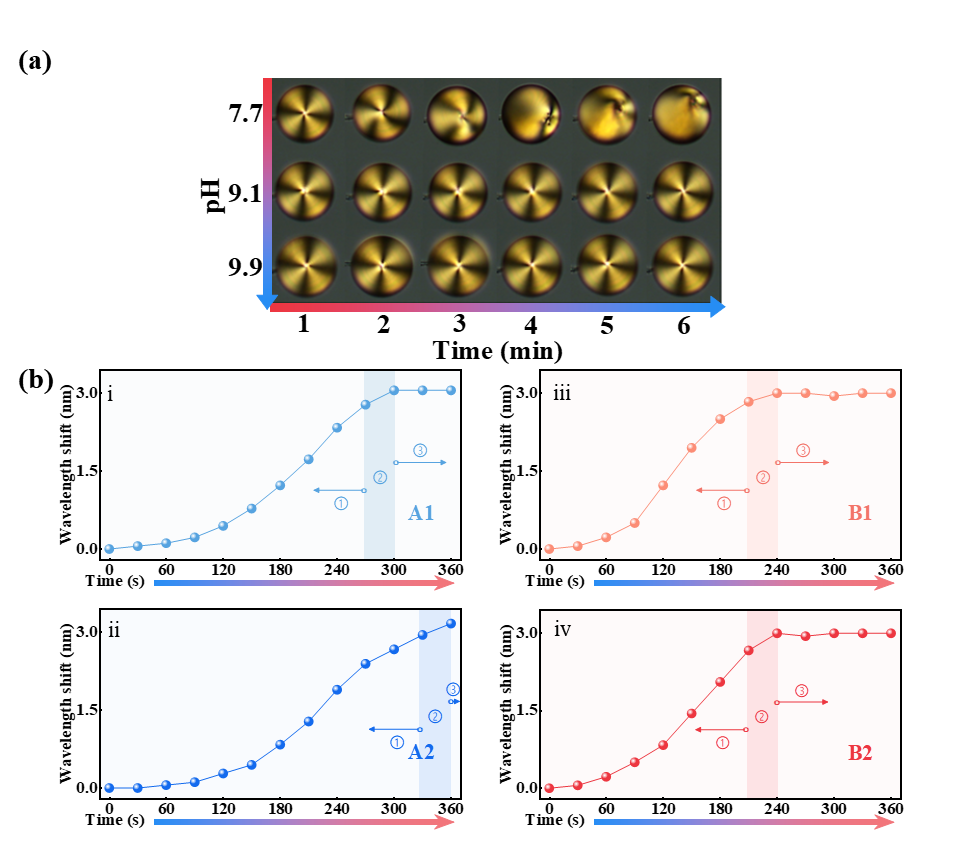


**Figure S12. (a)**The POM images of functionalized LC microcavities in buffer solutions (pH 7.7, 9.1 and 9.9) with mice serum samples **(b)** Wavelength shifts with different serum samples: A1, A2, B1, B2.


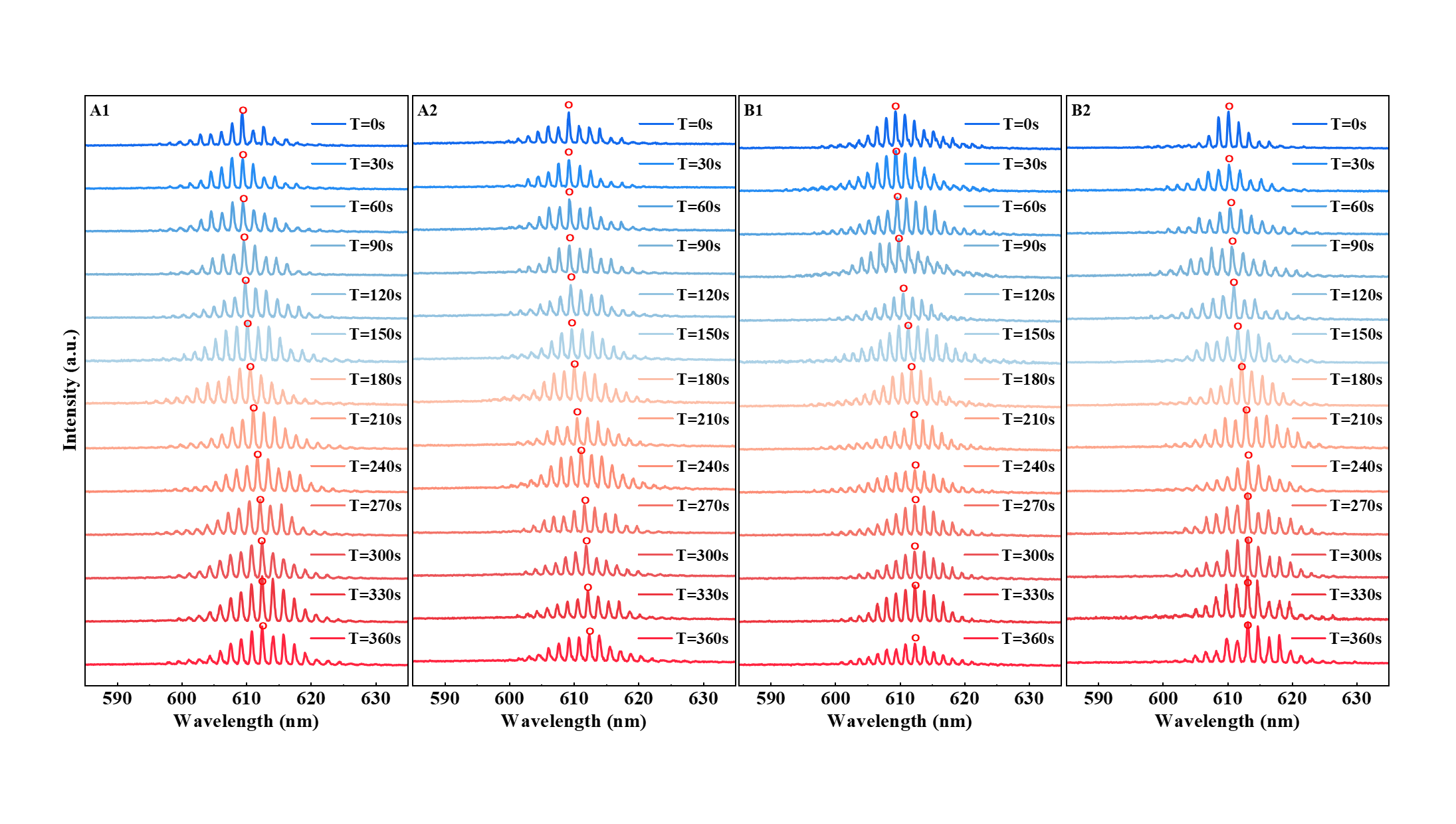


**Figure S13.** WGM spectra of ALT concentrations for serum samples: A1, A2, B1, B2.

**Table S1**. Comparison of ALT detection methods

| **Used Materials/Enzyme****/Substrate** | **Principle** | **Response**  **Time** | **Linear**  **Range** | **Reference** |
| --- | --- | --- | --- | --- |
| L-alanine, α-ketoglutarate, GMOx, [TBA]_2_[Ni(mnt)_2_]/graphene composite film, glutaraldehyde | Electrochemical | / | 5-140  (U/L) | 13 |
| L-alanine, α-ketoglutarate, TPP, FAD, pyruvate oxidase, MgCl₂, graphene@MXene, glutaraldehyde | Electrochemical | ~40 min | 5-400  (U/L) | 14 |
| molecular beacons 1 with FITC, molecular beacons 2 with TMR, Oligo1, Oligo2, E. coli DNA ligase, NAD^+^ assay buffer | Fluorescence | ~10 min | 0-3.13  (U/L) | 15 |
| L-alanine, α-ketoglutarate, lactate dehydrogenase, H–MnO₂, Sodium Alginate, Tris-HCl buffer, NADH | Colorimetric | ~30 min | 1-220  (U/L) | 16 |
| L-alanine, α-ketoglutarate, 2,4-dinitrophenyl hydrazine, Ca^2+^, NaOH | Colorimetric | ~50 min | 20-140  (U/L) | 17 |
| L-alanine, pyruvate oxidase, horseradish peroxidase, 4-aminoantipyrine, N, N-dimethylaminobenzoic acid | Colorimetric | ~15 min | 40-200  (U/L) | 18 |
| L-alanine, α-ketoglutarate, DC-ECL chip/detection layer, Luminol, pyruvate oxidase, MgCl₂, thiamine pyrophosphate | Electrochemiluminescence | ~10 min | 5-50  (U/L) | 19 |
| L-alanine, α-ketoglutarate, taper-in-taper structural SMF with AuNPs, MoS_2_-NPs, CeO_2_-NPs, GluOx, MUA, EDC, NHS | LSPR | ~3 min | 10-1000  (U/L) | 20 |
| L-alanine, α-ketoglutarate, 5CB with DCM and stearic acid | WGM | ~6 min | 0-240  (U/L) | This work |

**References**

1. Qu R, George TF, Li GL. Development in liquid crystal microcapsules: fabrication, optimization and application. J Mater Chem C. 2021; 10: 413–432.
2. Liu DD, Jang CH. A new strategy for imaging urease activity using liquid crystal droplet patterns formed on solid surfaces. Sensor Actuat B-Chem. 2014; 193: 770–773.
3. Humar M, Ravnik M, Pajk S, Muševič I. Electrically tunable liquid crystal optical microresonators. Nat Photonics. 2009; 3(10): 595–600.
4. Tian DZ, Xiang W, Wang H, Jiang WT, Li T, Yang MH. Optical assay using B‑doped core–shell Fe@BC nanozyme for determination of alanine aminotransferase. Microchim Acta. 2022; 189: 147.
5. Jakob BC, Silvia G, Laurent B, Olivier TG. Low-cost disposable ALT electrochemical microsensors for in-vitro hepatotoxic assessment. Sensor Actuat B-Chem. 2016; 228: 360–365.
6. Dong CH, He L, Xiao YF, Gaddam VR, Ozdemir SK, Han ZF, Guo GC, Yang L. Fabrication of high-Q polydimethylsiloxane optical microspheres for thermal sensing. Appl Phys Lett. 2009; 94: 231119.
7. Yang X, Tang SJ, Meng JW, Zhang PJ, Chen YL, Xiao YF. Phase-transition microcavity laser. Nano Lett. 2023; 23(7): 3048–3053.
8. Chen R, Ta VD, Sun HD. Bending-Induced Bidirectional Tuning of whispering gallery mode lasing from flexible polymer fibers. ACS Photonics. 2014; 1(1): 11–16.
9. Moon BS, Lee TK, Jeon WC, Kwak SK, Kim YJ, Kim DH. Continuous-wave upconversion lasing with a sub-10 W cm−2 threshold enabled by atomic disorder in the host matrix. Nat Commun. 2021; 12: 4437.
10. Duan R, Zhang ZT, Xiao L, Ren TH, Zhou XH, Thung YT, Ta VD, Yang J, Sun HD. Dome-shaped mode lasing from liquid crystals for full-color lasers and high-sensitivity detection. Chem Commun. 2023; 59(12): 1641–1644.
11. Jordan D, Charles WC, Peter RW. HetMM: A Michaelis-Menten model for non-homogeneous enzyme mixtures. IScience. 2024; 27(2): 108977.
12. Li JF, Hu HH, Chen XY, Zhu HT, Zhang WH, Tai ZY, Yu XD, He QY. A novel ACE inhibitory peptide from Douchi hydrolysate: Stability, inhibition mechanism, and antihypertensive potential in spontaneously hypertensive rats. Food chem. 2024; 460: 140734.
13. Jiang DF, Li CP, Liu T, Li LL, Chu ZY, Jin WQ, Ren XM. A regular nanostructured dithiolene metal complex film for ultrasensitive biosensing of liver enzyme. Sensor Actuat B-Chem. 2017; 241(31): 860–867.
14. Quan CY, Quan LL, Wen QY, Yang MH, Li T. Alanine aminotransferase electrochemical sensor based on graphene@MXene composite nanomaterials. Microchim Acta. 2024; 191: 45.
15. Tang ZW, Liu P, Ma CB, Yang XH, Wang KM, Tan WH, Lv XY. Molecular beacon based bioassay for highly sensitive and selective detection of nicotinamide adenine dinucleotide and the activity of alanine aminotransferase. Anal Chem. 2011; 83(7): 2505–2510.
16. Li SW, Chen ZH, Yang F, Yue WQ. Self-template sacrifice and in situ oxidation of a constructed hollow MnO_2_ nanozymes for smartphone-assisted colorimetric detection of liver function biomarkers. Anal Chim Acta. 2023; 1278: 341744.
17. Resmi P, Kumar SS, Alageswari D, Suneesh P, Ramachandran T, Nair B, Babu TS. Development of a paper-based analytical device for the colourimetric detection of alanine transaminase and the application of deep learning for image analysis. Anal Chim Acta. 2021; 1188: 339158.
18. Nira RP, Jason PR, Shailendra K, Patrick DB, Sidhartha J, Farzad N, Vicki LW, Rebecca AP, Una SR, George MW. A paper-based multiplexed transaminase test for low-cost, point-of-care liver function testing. Sci Transl Med. 2012; 4(152): 152ra129.
19. Lai W, Shi Y, Zhong J, Zhou X, Yang Y, Chen ZH, Zhang C. A dry chemistry-based electrochemiluminescence device for point-of-care testing of alanine transaminase. Talanta. 2023; 256: 124287.
20. Wang Z, Singh R, Marques C, Jha R, Zhang BY, Kumar S. Taper-in-taper fiber structure-based LSPR sensor for alanine aminotransferase detection. Opt Express. 2021; 29(26): 43793.
